# Natural mutations in the sensor kinase of the PhoPR two-component regulatory system modulate virulence of ancestor-like tuberculosis bacilli

**DOI:** 10.1371/journal.ppat.1011437

**Published:** 2023-07-14

**Authors:** Wladimir Malaga, Delphine Payros, Eva Meunier, Wafa Frigui, Fadel Sayes, Alexandre Pawlik, Mickael Orgeur, Céline Berrone, Flavie Moreau, Serge Mazères, Jesus Gonzalo-Asensio, David Rengel, Carlos Martin, Catherine Astarie-Dequeker, Lionel Mourey, Roland Brosch, Christophe Guilhot

**Affiliations:** 1 Institut de Pharmacologie et de Biologie Structurale (IPBS), Université de Toulouse, CNRS, Université Toulouse III – Paul Sabatier (UPS), Toulouse, France; 2 Institut Pasteur, Université Paris Cité, CNRS UMR6047, Unit for Integrated Mycobacterial Pathogenomics, Paris, France; 3 Grupo de Genética de Micobacterias, Facultad de Medicina, Departamento de Microbiologia, Pediatria, Radiologica y Salud Pùblica, Universidad de Zaragoza, Zaragoza, Spain; 4 CIBER Enfermedades Respiratorias, Institudo de Salud Carlos III, Madrid, Spain; 5 Servicio de Microbiologia, Hospital Universitario Miguel Servet, ISS Aragon, Zaragoza, Spain; National Institutes of Health, UNITED STATES

## Abstract

The molecular factors and genetic adaptations that contributed to the emergence of *Mycobacterium tuberculosis (MTB)* from an environmental *Mycobacterium canettii*-like ancestor, remain poorly investigated. In *MTB*, the PhoPR two-component regulatory system controls production and secretion of proteins and lipid virulence effectors. Here, we describe that several mutations, present in *phoR* of *M*. *canettii* relative to *MTB*, impact the expression of the PhoP regulon and the pathogenicity of the strains. First, we establish a molecular model of PhoR and show that some substitutions found in PhoR of *M*. *canettii* are likely to impact the structure and activity of this protein. Second, we show that STB-K, the most attenuated available *M*. *canettii* strain, displays lower expression of PhoP-induced genes than *MTB*. Third, we demonstrate that genetic swapping of the *phoPR* allele from STB-K with the ortholog from *MTB* H37Rv enhances expression of PhoP-controlled functions and the capacities of the recombinant strain to colonize human macrophages, the *MTB* target cells, as well as to cause disease in several mouse infection models. Fourth, we extended these observations to other *M*. *canettii* strains and confirm that PhoP-controlled functions are expressed at lower levels in most *M*. *canettii* strains than in *M*. *tuberculosis*. Our findings suggest that distinct PhoR variants have been selected during the evolution of tuberculosis bacilli, contributing to higher pathogenicity and persistence of *MTB* in the mammalian host.

## Author summary

*Mycobacterium tuberculosis* (*MTB*) likely emerged from an environmental ancestor by adaptation of endogenous pathways enhancing its capacity to colonize and transmit within the human host. The closest extant relatives of the *MTB* ancestor are the *Mycobacterium canettii* strains which are opportunist pathogens responsible for rare human tuberculosis cases in East-Africa. Here we found that the sensor protein of the important two-component regulatory system PhoPR displays amino acid substitutions that modulate its activity in *M*. *canettii* in comparison to *MTB*. These mutations impact the production and secretion of virulence factors important for multiplication within the host and cough induction. Therefore, our findings support the hypothesis that variants of the PhoPR regulatory system were selected as key evolutionary adaptations for enhancing the epidemic capacity of the tuberculosis bacilli.

## Introduction

*Mycobacterium tuberculosis (MTB)*, the etiological agent of human tuberculosis (TB), emerged from an environmental ancestor by step-wise adaptation of existing pathways and a few gene acquisitions by horizontal transfer [1–3]. Genomic analyses indicate that the closest extant relatives of this ancestor are the pool of *Mycobacterium canettii* strains [4–6], which are also known as smooth tuberculosis bacilli (STB) (S1 Fig). After the separation from the *M*. *canettii*-like ancestor, clonal evolution led to the *MTB* complex (MTBC) which gathers 8 principal phylogenetic lineages of *MTB* and *Mycobacterium africanum* strains that primarily infect humans, and an additional group of animal-adapted lineages [7,8]. Concerning *M*. *canettii*, around 100 TB cases caused by these strains have been recorded up to now which contrast to the 10 million annual cases due to MTBC strains [9]. Most patients infected by *M*. *canettii* have had links to the geographical region of the Horn of Africa, suggesting a yet unknown, potential environmental reservoir in this area [5,10]. Remarkably, no human-to-human transmission has been reported for *M*. *canettii* strains [11], indicating that these isolates do not exhibit the required genetic traits for efficient colonization and/or transmission in the human host. Consistently, *M*. *canettii* strains exhibit lower virulence than *MTB* in various human cellular and animal models [6,12,13]. An important question to understand the emergence of *MTB* is therefore the nature of the adaptation, genetic and phenotypic, that contributed to increase the *MTB* virulence since the branching from a *M*. *canettii*-like progenitor.

In previous studies, we identified two key events that likely favored the evolution of an *M*. *canettii*-like progenitor toward a strict pathogenic life style [14,15]. First, we found that an ancestral change in the cell surface composition associated with the inactivation of a lipooligosaccharide (LOS) biosynthesis pathway led to an enhanced capacity to multiply in susceptible cellular and animal models [14]. Second, we demonstrated that the *MTB* ancestor evolved by enhancing its resistance to stress, such as nitric oxide, to promote its persistence during the chronic phase of the infection [15]. In parallel, Chiner-Oms *et al*. [16] used bioinformatics analyses to identify 53 genomic regions that evolved under different selective pressures before and after the transition to obligate pathogens. Among them, *phoR*, was the only gene to evolve under positive selection in MTBC but not in *M*. *canettii* [16]. PhoR is the sensor kinase of the PhoPR two-component regulatory system required for full virulence of *MTB* [17–20]. PhoPR controls either directly or indirectly more than 80 genes of *MTB*, including the *espACD* operon required for the ESX-1-mediated secretion of the major virulence factor EsxA (also known as ESAT-6) as well as biosynthetic genes of surface lipids, such as sulfoglycolipids (SGL) or di- and poly-acyltrehaloses (DAT/PAT) [18,21–24].

Our objectives for this study were to explore the impact of PhoR polymorphisms on PhoP-controlled functions and on virulence as revealed in various infection models.

## Results

### Several mutations found in PhoR variants from *M*. *canettii* are located in catalytic domains of PhoR

**Fig 1:**
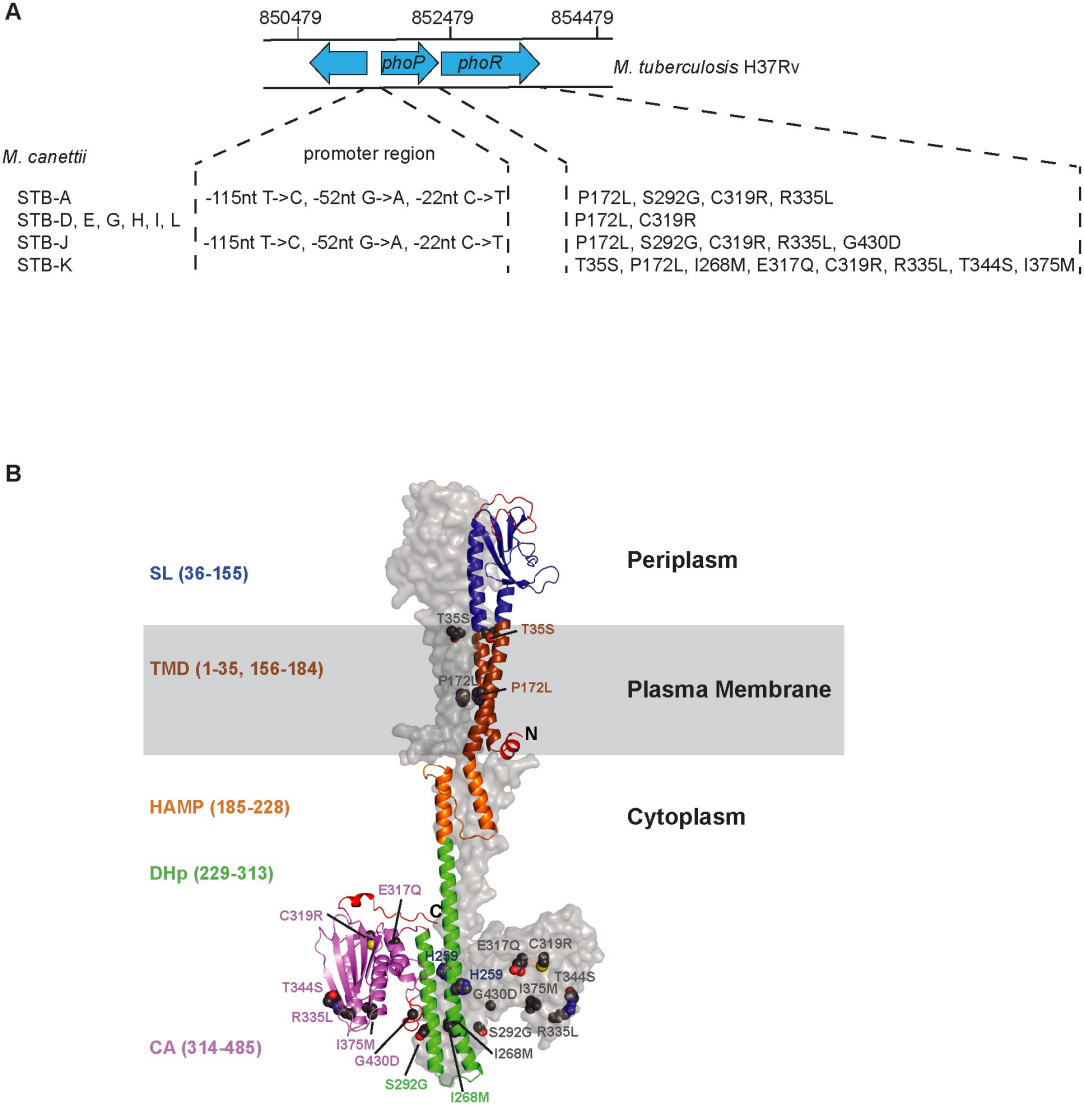
The mutations in PhoR variants from *M*. *canettii* impact the catalytic domain of PhoR. A) Position of promotor mutations and amino acid substitutions in PhoPR from *M*. *canettii* strains. The organization of the *phoPR* region and its location on the *MTB* H37Rv genome sequence are indicated. Only SNPs located within the promotor region of *phoPR* [31], the indicated position is relative to the *phoP* start codon, or SNPs changing the amino acid sequence of PhoR in comparison to the *H37Rv* proteins are mentioned. B) AlphaFold-Multimer predicted structure of the *MTB* PhoR dimer. One PhoR protomer is displayed in cartoon with sensor loop (SL) in blue, transmembrane helices (TMD) in brown, signal-transducing domain (HAMP) in orange, dimerization and histidine phosphotransfer (DHp) domain in green, and catalytic/ATP-binding domain (CA) in violet. Regions of low confidence (pLDDT < 70) are in red. Putative boundaries of the domains are given. The molecular surface of the other protomer is shown in grey. Mutated residues in the different *M*. *canettii* alleles are shown as spheres and labelled on both protomers. Figure generated using PyMOL (PyMOL Molecular Graphics System, Version 2.4.1 Schrödinger, LLC.).

Sequence comparison of *phoPR* genes identified several single-nucleotide polymorphisms (SNPs) in the tested *M*. *canettii* strains (named STB-A, D, E, G, H, I, J, K and L) in comparison to *MTB* H37Rv. The closest sequence was that of STB-L, which exhibits 6 nucleotide differences to that of *MTB* H37Rv, all of them located in *phoR*. The other *M*. *canettii* isolates analyzed revealed a higher number of SNPs (up to 36 SNPs for STB-K). However, most of them are synonymous. Remarkably, all *M*. *canettii* strains exhibit a PhoP amino-acid sequence 100% conserved with that of PhoP from H37Rv. In contrast, PhoR from *M*. *canettii* strains were more diverse with 2 to 8 amino acid substitutions in comparison to PhoR from H37Rv (Fig 1). One of these mutations, P172L was specific for the H37Rv reference strain [25] and absent from most *MTB* genomes screened. The others were specific for each of the *M*. *canettii* strains.

PhoR comprises 485 amino acid residues and is anchored to the plasma membrane by two 30-residue long transmembrane helices (TMD) separated by a 120-residue periplasmic sensor segment, often referred to as sensor loop (SL) [24]. The remaining part of the protein is located in the cytosol and contains an intracellular signal-transducing domain (HAMP, ≈50 residues) followed by the dimerization and histidine phosphotransfer domain (DHp, ≈80 residues), which bears the phosphorylation site histidine, and the catalytic/ATP-binding (CA, ≈170 residues) domain [24]. The only reported structure about *MTB* PhoR is that of the DHp domain (residues 240–310) resolved using X-ray crystallography at 1.9 Å resolution (PDB 5UKY) [26].

To further outline the 3D architecture of PhoR, the PhoR amino acid sequence was submitted to RoseTTAFold [27] at https://robetta.bakerlab.org/ and the resulted prediction was compared to the prediction retrieved from the AlphaFold Protein Structure Database [28] at https://alphafold.ebi.ac.uk/entry/P71815. Both algorithms predicted the PhoR tertiary structure with high confidence, with approx. 90% of residues having pLDDT > 70. Regions of low confidence (residues pLDDT < 70) are located at the N and C termini of the protein and in three loops (Figs 1B and S2). However, overall superimposition of the two predicted models led to a rmsd of 7.7 Å based on the Cα atoms (S2A Fig) whereas per-domain superimposition gave impressive rmsd values, between 0.9 and 2.0 Å (S2B Fig), indicating that the spatial relationship of individual domains cannot be unambiguously predicted using current prediction tools [29]. Since PhoR is expected to form a homodimer, we tried to generate its quaternary structure using AlphaFold-Multimer [30]. The PhoR dimer generated (Fig 1B) contained only very few “bad” intra- and inter-protomer contacts and superposing the DHp domain of the predicted full-length PhoR dimer and the X-ray structure of the PhoR DHp dimer revealed a rmsd value of 1.4 Å with the highest deviations located at both extremities of the α1 and α2 helices (S3 Fig), especially in one of the protomers, in line with the observed asymmetry in the DHp crystal structure [26].

We next positioned the amino-acids substitutions found in PhoR variants from *M*. *canettii* and analyzed their chemical environment (Fig 1B and S1 Text). Our results strongly suggest that some of these changes may affect the stability and activity of PhoR (Figs 1B and S4 and S1 Text).

### The PhoPR-positively regulated genes are underexpressed *in vitro* and *in vivo* in *M*. *canettii* STB-K

To further investigate the impact of *phoR* polymorphisms in *M*. *canettii*, we first focused on STB-K as a model because PhoR from STB-K (PhoR-STB-K) contains 8 amino acid changes, the largest number among the *M*. *canettii* strains, in comparison to that of H37Rv (PhoR-H37Rv), with 2 or 3 of them common to other *M*. *canettii* strains (Fig 1A).

**Fig 2:**
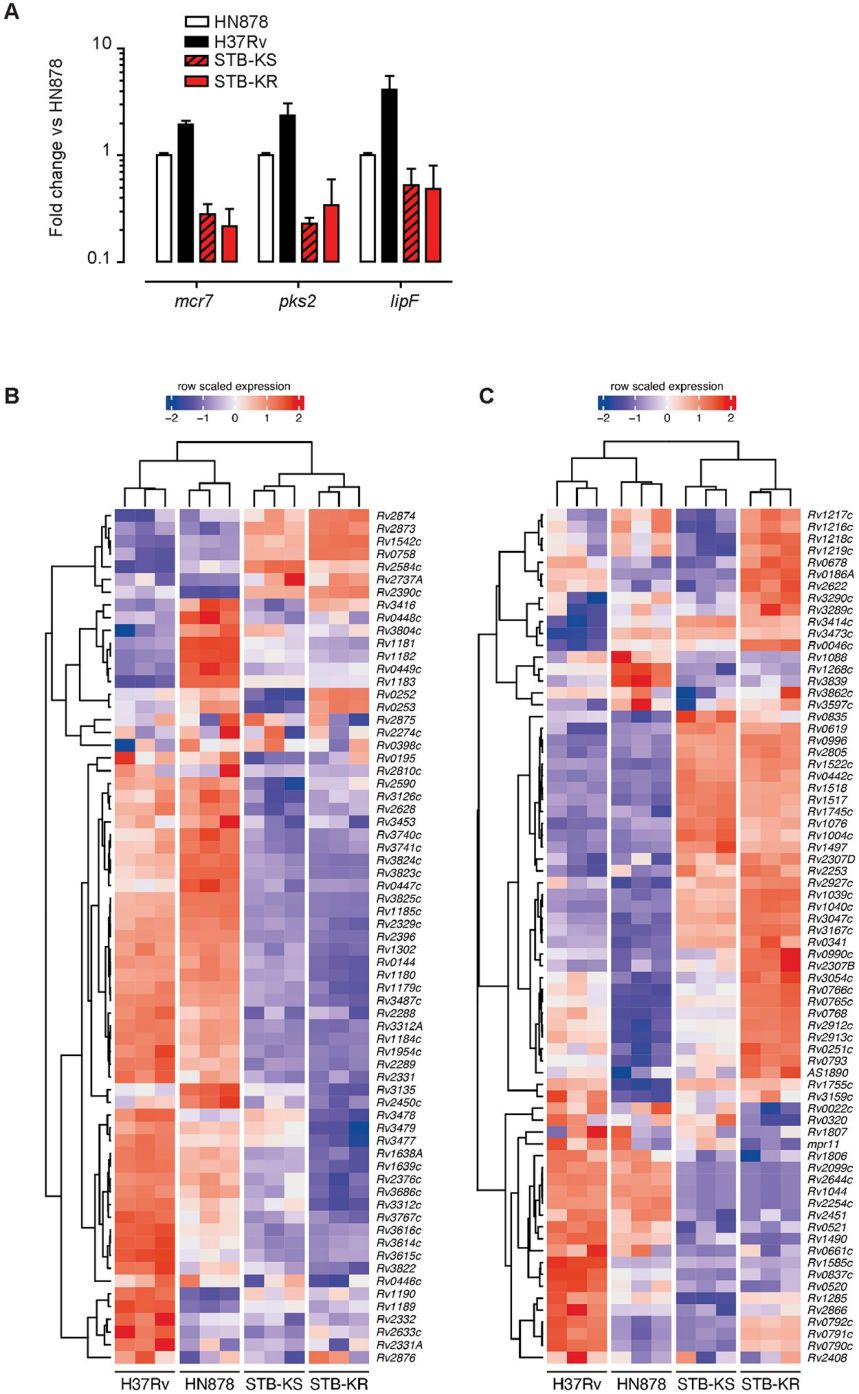
PhoPR-regulated genes are expressed at higher levels in *MTB* H37Rv or HN878 strains than in *M*. *canettii* STB-KS or STB-KR strains grown *in vitro*. A) RT-qPCR analysis of expression of 3 genes from the PhoP regulon. Bars represent fold changes in the expression levels of three genes relative to HN878 in strains H37Rv and two *M*. *canettii* STB-K isolates with morphotype smooth (KS) or rough (KR). The value for each gene was normalized over the housekeeping gene *sigA*. The presented results are means +/- SD of 3 independent experiments performed in triplicate. B, C) Expression levels of the genes regulated by PhoP in *MTB*, H37Rv and HN878, and both *M*. *canettii* STB-K isolates. Depicted genes correspond to the positively-regulated (B) and negatively-regulated genes (C) according to [23]. Gene expression levels were calculated following normalization and regularized logarithm transformation of raw read counts determined by RNAseq.

We employed RT-qPCR to examine the expression of genes from the PhoP-regulon in two morphotypes of STB-K, STB-KS (smooth) and STB-KR (rough), which differ by their production of LOS [14] and two *MTB* strains, HN878 and H37Rv, from phylogenetic lineages 2 and 4 respectively. We selected three genes tightly positively-controlled by PhoPR in *MTB* [18,23], *lipF*, *pks2* and the non-coding RNA *mcr7*, and their expression was normalized against the constitutively expressed *sigA* gene as a housekeeping control. We found 2 to 4 fold reduced amounts of mRNA for the 3 genes in STB-KS and STB-KR in comparison to the two *MTB* strains (Fig 2A). We also observed a higher expression of the three genes in H37Rv compared to HN878 that ranged between 1.9 and 4.1 fold.

To extend these observations on a wider range of genes from the PhoP-regulon, we performed RNAseq on RNA preparations that were extracted from the four strains, grown to exponential phase in standard 7H9 medium. We found that most PhoP-positively regulated genes are expressed at higher level in H37Rv and HN878 than in STB-KS and STB-KR (Figs 2B and S5A). The situation is more contrasted for the genes negatively regulated by PhoP (Figs 2C and S5B) because half of them were less expressed in the two *MTB* vs the two STB-K strains whereas the others were more expressed. One possible explanation is that the original list of differentially expressed genes was generated by comparing the *phoP* mutant against the parental H37Rv strain [18,23], while we compared here WT strains expressing identical PhoP but PhoR variants. Therefore, the difference may reflect the impact of PhoR-mediated phosphorylation on PhoP on gene repression. An additional parameter might be related to the different genetic background of the strains (S1 Fig), as reflected by the higher number of differentially expressed genes detected in both STB-K morphotypes than in *MTB* HN878 in comparison to H37Rv (1113 and 1103 vs 469, S1 Table). In any case, there is a clear difference in the expression profiles of PhoPR-regulated genes between the two *MTB* vs the two STB-K strains in our RNAseq analysis and these results support the hypothesis that the PhoPR control is stronger in *MTB* than in STB-K.

In conclusion, these results indicate that the genes positively controlled by PhoP are underexpressed in *M*. *canettii* STB-K in comparison to *MTB* in standard laboratory growth conditions.

### Mutations in the *phoPR* allele from *M*. *canettii* STB-K impact functionality

**Fig 3:**
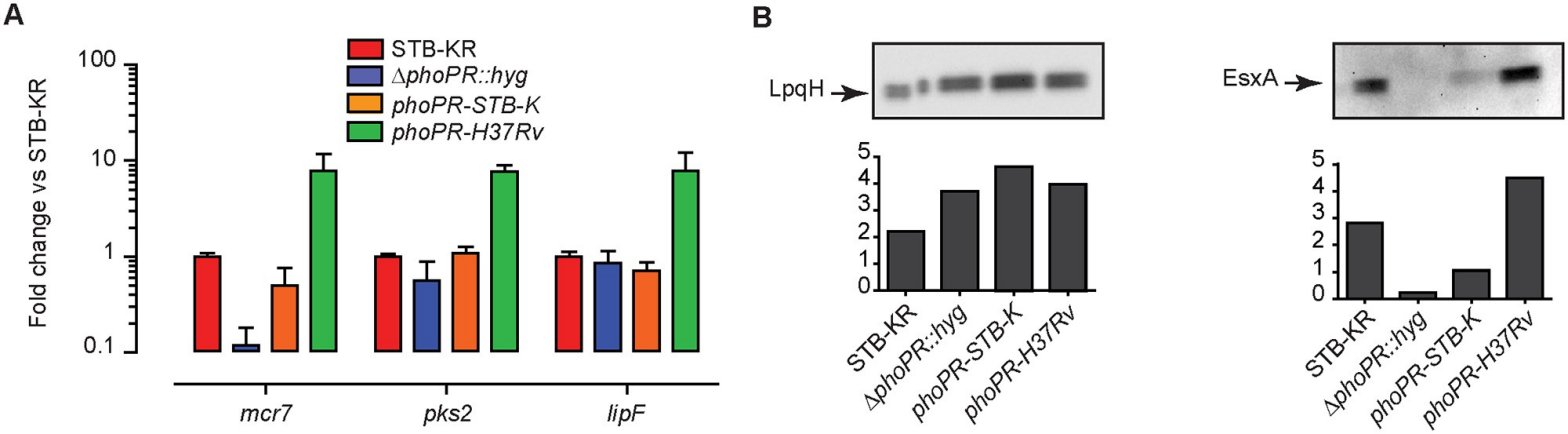
Expression of *phoPR-H37Rv* in *M*. *canettii* STB-KR increases expression of PhoP-regulated genes and secretion of EsxA. A) RT-qPCR analysis of expression of 3 genes from the PhoP regulon in the *M*. *canettii* STB-KR, Δ*phoPR*::*hyg* mutant, and *phoPR-H37Rv* or *phoPR-STB-K* complemented strains. Bars represent fold changes in the expression levels of three genes relative to *M*. *canettii* STB-KR wild-type. The presented results are means +/- SD of 3 independent experiments performed in triplicate. B) Western blot analysis of proteins from the culture supernatants of *M*. *canettii* STB-KR, Δ*phoPR*::*hyg* mutant, and *phoPR-H37Rv* or *phoPR-STB-K* complemented strains. The protein extracts were probed with EsxA- or LpqH-specific antibodies. Signal intensity (arbitrary unit) was plotted in the graph below the Western-blot image. The presented results are representative of 2 independent experiments.

We next investigated whether mutations in the *phoPR* genes were responsible for this lower expression of the PhoPR-positively regulated genes in *M*. *canettii* STB-K. To this end, we constructed a series of recombinant strains derived from STB-KR. We first produced a mutant strain, STB-KR *ΔphoPR*::*hyg* (S6 Fig). Next, we transferred integrative vectors carrying either the STB-K allele of the *phoPR* genes (*phoPR-STB-K*) or the H37Rv allele (*phoPR-H37Rv*) into this mutant. Then we compared the expression of *lipF*, *pks2*, *mcr7* in the various strains (Fig 3A). We found that disruption of the *phoPR* genes in STB-KR reduced the expression of these three genes *in vitro* (9x for *mcr7*, 2x for *pks2* and 1.2x for *lipF*), as in *MTB* H37Rv [18,23]. Transfer of the *phoPR-STB-K* allele in the STB-KR *ΔphoPR*::*hyg* mutant restores the expression of the three genes to a level slightly lower or similar to that in STB-KR. In sharp contrast, the recombinant strain expressing the *phoPR-H37Rv* allele displayed a strongly increased expression of the three genes, *mcr7*, *pks2*, and *lipF* with 7–15 fold higher transcript levels respectively in comparison to the strain expressing *phoPR-STB-K* (Fig 3A). We found a slightly higher expression of the *phoP* and *phoR* genes in the STB-KR *ΔphoPR*::*hyg* mutant complemented with *phoPR-STB-K* than in the isogenic strain expressing *phoPR-H37Rv* (S7 Fig), consistent with a negative feedback regulatory loop as observed previously [31]. Under acidic condition known to induce PhoP-positively regulated genes [32], we also observed a higher expression of *mcr7* in the recombinant strain expressing *phoPR-H37Rv* in comparison to the isogenic strain expressing *phoPR-STB-K*, although the difference was lower than at neutral pH (Figs 3A and S8).

This higher expression of PhoPR-positively controled genes in the recombinant strain expressing *phoPR-H37Rv* correlates with higher secretion of EsxA. Indeed, while we observed similar amounts of LpqH (Fig 3B), a protein whose production and secretion is not controlled by PhoPR, we found that the expression of *phoPR-H37Rv* in the Δ*phoPR*::*hyg* mutant substantially increased EsxA secretion relative to the isogenic strain expressing *phoPR-STB-K*. Of note, a partial restoration of EsxA secretion was obtained for the Δ*phoPR*::*hyg* strain complemented with *phoPR-STB-K* consistent with the RT-qPCR results.

As EsxA is a major antigen whose secreted form is strongly recognized by host immune cells [22,33,34], we also compared the induction of ESX-1 antigen-specific T-cell responses in mice infected with *MTB* H37Rv, H37Rv ΔESX-1 (used as a control deficient for EsxA secretion), STB-KR, the STB-KR *ΔphoPR*::*hyg* mutant, and the STB-KR *ΔphoPR*::*hyg* mutant complemented with the *phoPR*-STB-K or *phoPR-H37Rv* (S9 Fig). Interestingly, this experiment showed that splenocytes from mice infected with STB-KR, the *ΔphoPR*::*hyg* mutant or the mutant complemented with *phoPR-STB-K* only recognized antigens Ag85A, PE19 and PPE25 but not EsxA, EsxB and EspC. In contrast, the mutant complemented with the *phoPR-H37Rv* allele did induce similar ESX-1-specific T-cell responses as H37Rv. The ability of mycobacterial strains to induce these types of specific T-cell responses against secreted mycobacterial antigens in mice is usually strictly linked to the ability of the *MTB* strains to not only express the proteins but also to actively secrete them outside the bacterial cell [22,33,34]. Secretion of Ag85A is mediated by the Twin Arginine Translocation (TAT) protein secretion system whose expression is modulated indirectly by PhoPR in *MTB* through the ncRNA *mcr7* [23]. However, the observation that Ag85A-specific T cell responses are induced upon infection by all the tested strains, including *MTB* H37Rv and *M*. *canettii* STB-KR, suggests that the modulation of *mcr7* expression does not cause an on-off effect on Ag85A secretion, and that enough antigen might be present under *in vivo* conditions to induce T-cell responses even in strains expressing an active PhoPR system [23].

Taken together these results established that the *phoPR-STB-K* allele is deficient in comparison to the *phoPR-H37Rv* allele and that the PhoPR-positively regulated genes are underexpressed *in vitro* and *in vivo* in *M*. *canettii* STB-KR.

### The *phoPR-STB-K* allele confers lower virulence to *M*. *canettii* STB-KR than *phoPR-H37Rv* in human monocytes-derived-macrophages (hMDM)

**Fig 4:**
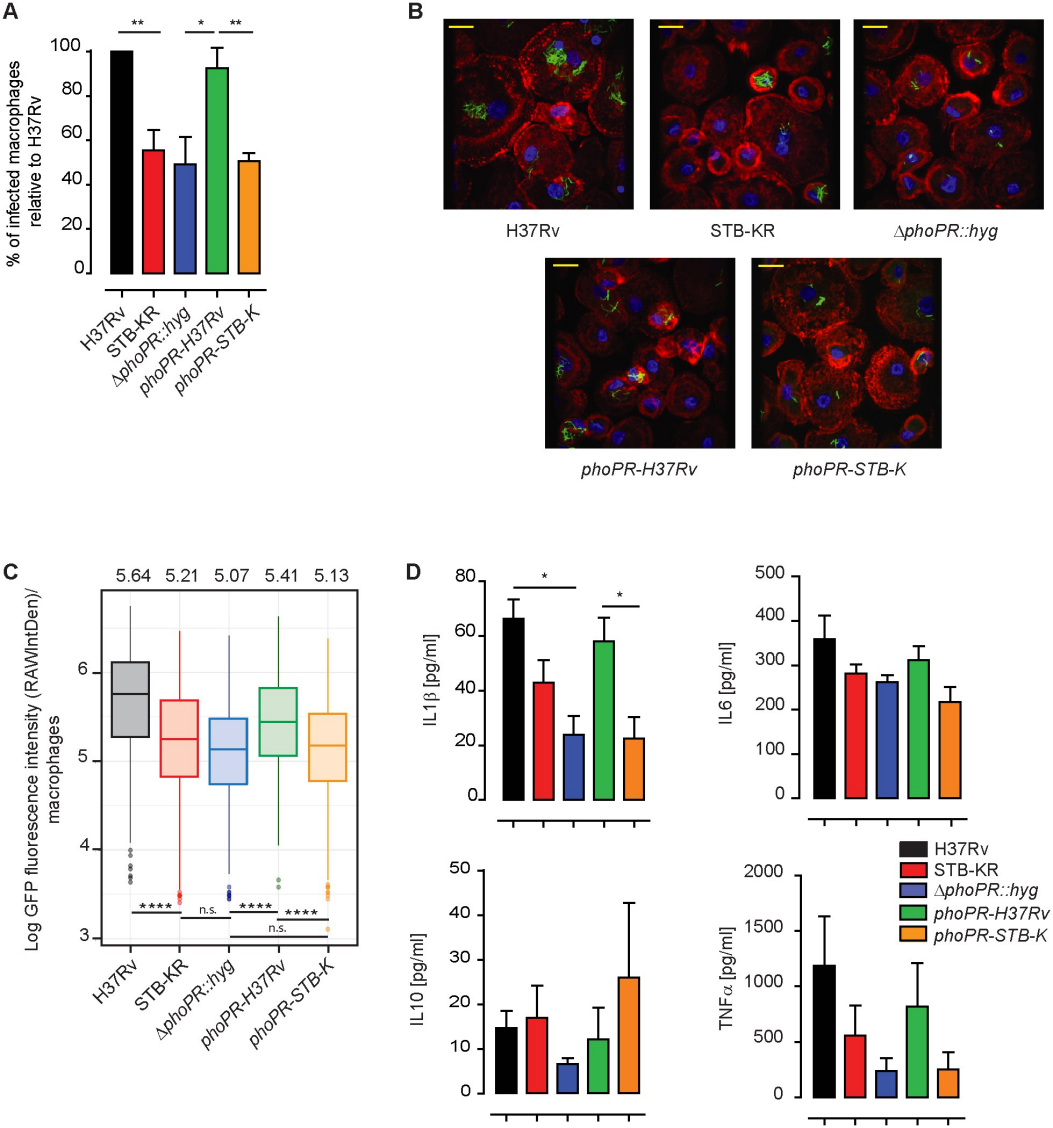
The *phoPR-H37Rv* allele confers higher infectivity to *M*. *canettii* STB-KR than *phoPR-STB-K* in human monocytes-derived-macrophages. A) To evaluate the capacity of H37Rv, STB-KR, STB-KR *ΔphoPR*::*hyg* and recombinant STB-KR strains expressing *phoPR-H37Rv* or *phoPR-STB-KR* to infect human macrophages, hMDMs were infected at a MOI of 10 bacteria per cell for 1 h. Cells were washed, fixed, and the percentage of infected cells was evaluated. More than 100 cells per donor from different fields were analyzed for each sample to measure the percentage of infected cells. Vertical bar plots indicated the percentage of hMDMs having ingested at least one bacterium relative to the situation for H37Rv set to 100%. The values are means +/- SEM calculated from 5 donors. The difference between the experimental groups was evaluated by one-way ANOVA and Bonferroni’s comparison test values. P values, * P<0.05, ** P<0.01. B) Representative images of hMDMs infected for 168 h with the various strains (nuclei are stained in blue, actin in red and bacteria in green). Scale bar, 10 μM. C) To monitor the intracellular growth of H37Rv, STB-KR, STB-KR *ΔphoPR*::*hyg* and recombinant STB-KR strains expressing *phoPR-H37Rv* or *phoPR-STB-KR*, hMDM were infected at a MOI of 2 bacteria per cell for 2 h. After infection, cells were washed and further incubated in the presence of serum. At 2 h and 168 h post-infection, the fluorescence intensity (RAWIntDen) per infected cell was measured. For each lot of hMDM (derived from 5 different donors), 50 to 140 infected cells were analyzed. Box plot of the Log fluorescence intensity (RAWIntDen) measured for each strain at 168 h post-infection (the value for individual hMDM from each donor and a cluster analysis is provided in S10 Fig). For each strain, the mean value was also calculated and is indicated above the graph. The difference between the experimental groups was evaluated using a mixed linear models and related post-hoc tests. P values, **** p<0.0001, n.s. non significative, p>0.05. D) IL1β, IL6, IL10 and TNFα production by hMDMs after infection with *MTB*, *M*. *canettii* STB-KR, and STB-KR-derived strains. Cells were infected at MOI 2 for 2 h, washed and incubated with serum containing medium. 2 days post-infection cell supernatants were collected, filtered and the level of cytokines was determined by ELISA. These experiments were performed with hMDMs derived from four or five different donors. The difference between the experimental groups was evaluated by one-way ANOVA and Bonferroni’s comparison test values. P values, * P<0.05.

The expression of a functional *phoPR* allele correlates with production and/or secretion of several key virulence factors, such as EsxA, in *MTB*. We therefore compared the virulence of the series of strains derived from STB-KR in macrophages, the main target cells of *MTB*. To this end, we transferred into these strains a plasmid expressing GFP. We incubated hMDMs for 1 h with *MTB* H37Rv, STB-KR, the STB-KR Δ*phoPR*::*hyg* mutant and the mutant complemented with either *phoPR-H37Rv* or *phoPR-STB-K* at a multiplicity of infection (MOI) of 10 bacteria per cell. Immediately after infection, we found that the percentage of infected cells was significantly lower for STB-KR and the STB-KR Δ*phoPR*::*hyg* mutant than for *MTB* H37Rv. Complementation with *phoPR-STB-K* did not change the percentage of infected cells in comparison to STB-KR in contrast to the complementation with *phoPR-H37Rv*, which significantly enhanced the infectivity to reach a similar level than that observed for H37Rv (Fig 4A).

We next compared the intracellular fate of these strains. To this end, hMDMs were infected with GFP-expressing strains at a MOI of 2 for 2 h at 37°C. At 2 h or 168 h post-infection, we assessed the percentage of infected cells as well as their fluorescence intensity (reflecting the bacterial load) (Figs 4B and 4C and S10). At 2 h post-infection, we observed a lower percentage of infected cells for STB-KR and the Δ*phoPR*::*hyg* mutant than for H37Rv and for the *phoPR-STB-K* complemented strain than for the isogenic *phoPR-H37Rv* complemented strain (S10A Fig). These results confirmed the difference observed initially at a different time of infection and MOI (Fig 4A). In contrast, the fluorescence intensity in infected cells was similar for all the strains (S10B–10D Fig). At 168 h post-infection, we still observed a higher percentage of infected cells for H37Rv than for STB-KR and STB-KR Δ*phoPR*::*hyg* and for the complemented strain expressing *phoPR-H37Rv* in comparison to the isogenic strain expressing *phoPR-STB-K* (S10A Fig). We also measured a higher bacterial load for H37Rv vs STB-KR and vs the Δ*phoPR*::*hyg* mutant and for the complemented strain expressing *phoPR-H37Rv* in comparison to that expressing *phoPR-STB-K* (Figs 4B and 4C and S10E and S10F).

We also monitored the inflammatory responses of infected hMDMs. As shown in Fig 4D, macrophages infected with strains expressing *phoPR-H37Rv* produced significantly more IL1β and a trend was also observed for IL6 and TNFα.

Thus, all these results indicate that expression of *phoPR-H37Rv* in *M*. *canetti* STB-KR increases bacterial virulence in hMDMs and induces a higher inflammatory response in comparison to the isogenic recombinant strain expressing *phoPR-STB-K*.

### The *phoPR-H37Rv* allele confers higher virulence to *M*. *canettii* STB-K than its own allele in mouse models

**Fig 5:**
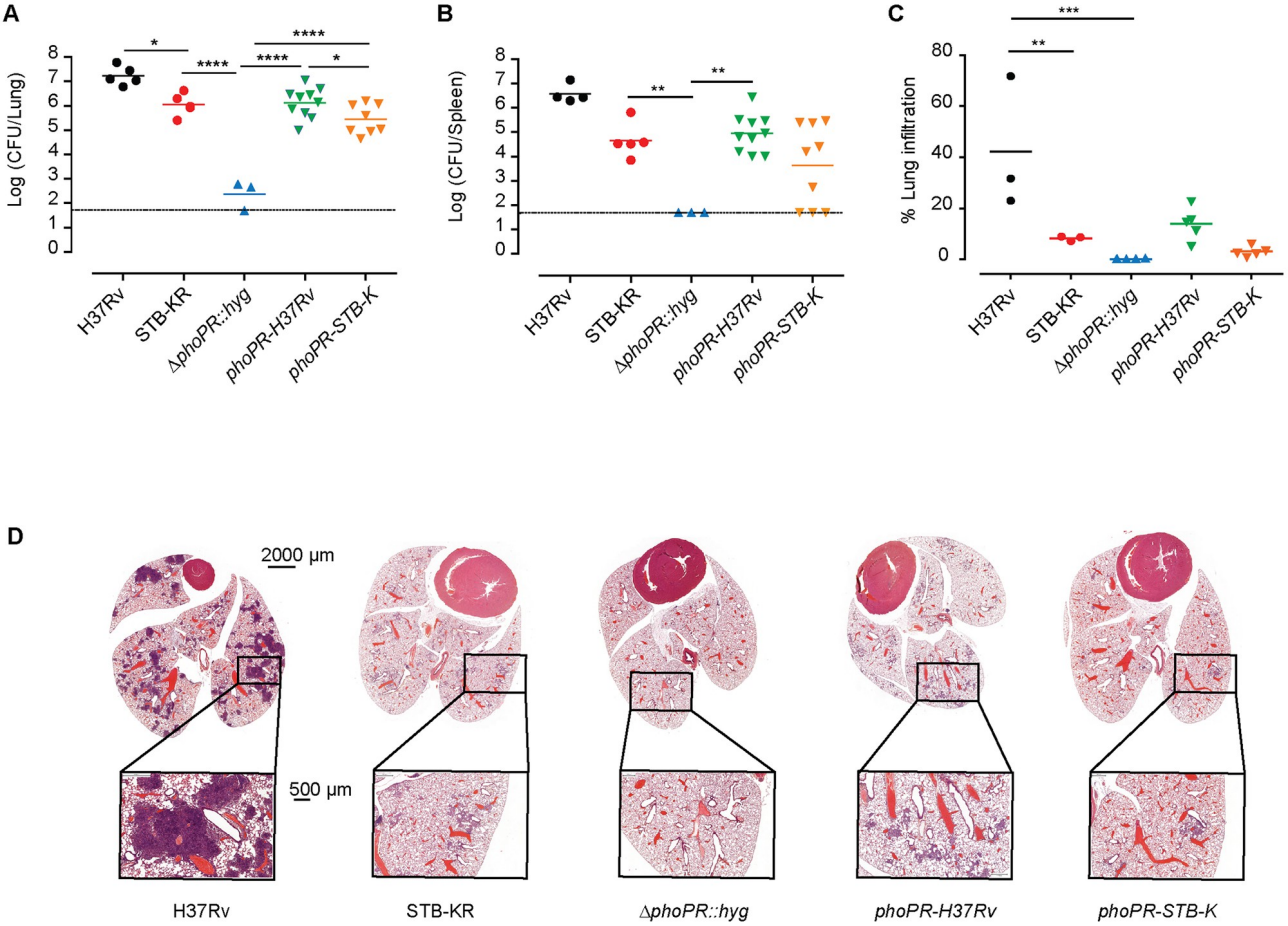
Expression of *phoPR-H37Rv* in *M*. *canettii* STB-KR enhances virulence in the C3HeB/FeJ mouse model. A, B) 5 groups of mice were infected intravenously with approximately 5x10^2^ cfu (10^2.5^ for STB-KR, 10^2.9^ for H37Rv, 10^2.2^ for STB-KR *ΔphoPR*::*hyg*, 10^2.4^ for *ΔphoPR*::*hyg*::*phoPR-H37Rv and* 10^2.7^ for *ΔphoPR*::*hyg*::*phoPR-STB-K)*. Bacterial loads were evaluated in the lungs (A) and spleen (B) 84 days post-infection. The presented results are representative of three independent experiments. The difference between the experimental groups was evaluated by one-way ANOVA and Bonferroni’s comparison test values. P values, * P<0.05, ** P<0.01, *** P<0.001, **** P<0.0001. C, D) For histopathology analysis, five groups of mice were infected with approximately 10^3^ cfu (10^3.9^ for STB-KR, 10^3.9^ for H37Rv, 10^3.3^ for STB-KR *ΔphoPR*::*hyg*, 10^3.7^ for *ΔphoPR*::*hyg*::*phoPR-H37Rv and* 10^3.6^ for *ΔphoPR*::*hyg*::*phoPR-STB-K)*. The lungs were recovered from the infected mice 70 days post-infection, fixed in 4% formalin and embedded in paraffin. Mid-lung sections were stained with hematoxylin and eosin and analyzed. C) Area of lung infiltration for each section. Each dot corresponds to one mouse. The difference between the experimental groups was evaluated by one-way ANOVA and Bonferroni’s comparison test values. P values, ** P<0.01, *** P<0.001. D) Representative images of lung sections from mice infected with different strains. Entire organ, scale = 2000 μM; window, scale = 500 μM.

To investigate further the effect of the expression of *phoPR-H37Rv* in STB-KR, we compared the virulence of our series of strains in the C3HeB/FeJ mouse model, which reproduces more closely the tissue lesions seen in TB patients [35]. Five groups of mice were infected intravenously with approximately 10^3^ colony-forming units (cfu) of the various strains. First, the bacterial load was evaluated in lungs and spleen 84 days post-infection (Fig 5A and 5B). We found that the STB-KR was significantly attenuated in comparison to *MTB* H37Rv in this murine infection model. The Δ*phoPR*::*hyg* mutation further attenuated the STB-KR Δ*phoPR*::*hyg* strain and the bacterial load was found below detection level in spleens (corresponding to ~1.7 log cfu) and more than 3 log lower than the parental strain in the lungs. Complementation with either *phoPR-H37Rv* or *phoPR-STB-K* both significantly increased the virulence of the recombinant strains but the bacterial load was significantly higher for the strain expressing *phoPR-H37Rv* in comparison to *phoPR-STB-K* (0.7 log higher in the lungs and 1.3 log higher in the spleen). Of note, expression of the *phoPR* allele from STB-K *via* the integrated complementation plasmid did not provide exactly the same level of virulence as the parental WT STB-KR strain. This partial complementation obtained upon expression of *phoPR* from an integrative plasmid was observed previously [18,36], and may suggest that the genomic position of the integrated *phoPR* allele may play some role to achieve a physiological expression of the *phoPR* operon.

Histopathological analyses showed a stronger inflammation in the lungs of mice infected with H37Rv than in the group infected with STB-KR (Fig 5C and 5D): the surface of inflammatory lesions was higher for H37Rv than for STB-KR and there was a trend indicating also a higher number of lesions for the *MTB* strain (S11 Fig). Consistent with the low bacterial load of the Δ*phoPR*::*hyg* mutant, inflammatory lesions were barely detectable in mice infected with this strain. Finally, comparison of lung lesions in mice that were infected with *phoPR-STB-K* or *phoPR-H37Rv* complemented strains showed a trend for both a higher number and size of inflammatory lesions in the *phoPR-H37Rv* complemented group (Figs 5C and 5D and S11).

**Fig 6:**
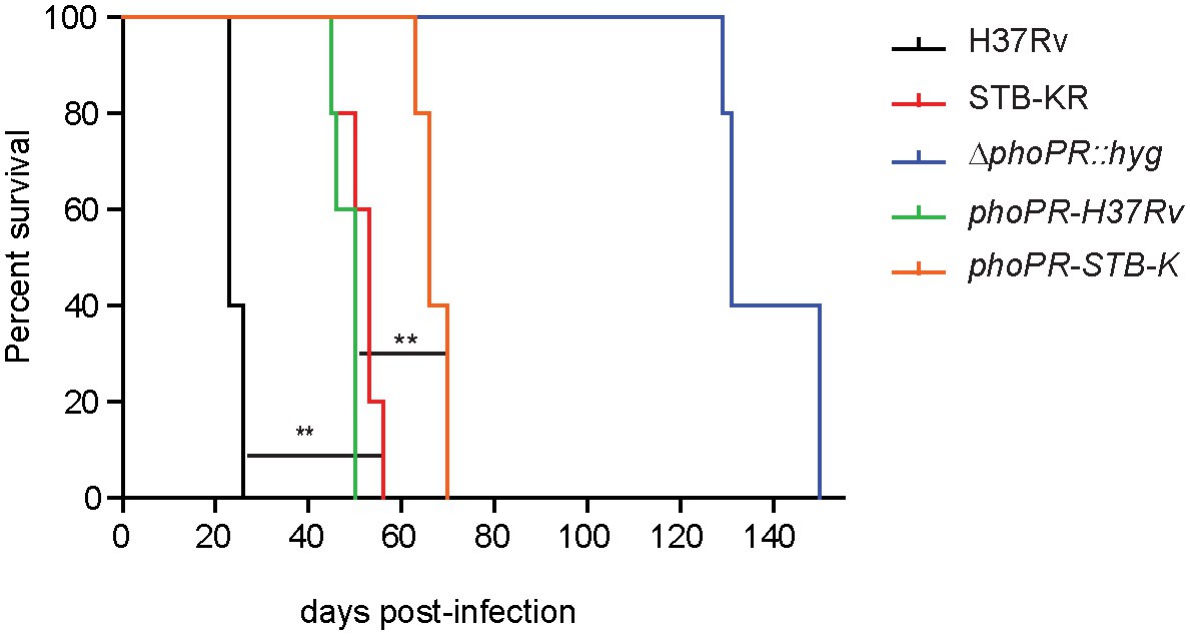
Expression of *phoPR-H37Rv* in *M*. *canettii* STB-KR enhances virulence in immunocompromised mice. Five groups of five SCID mice were infected intranasally with approximately 10^3^ cfu per mouse (10^3.2^ for STB-KR, 10^3.3^ for H37Rv, 10^3.1^ for STB-KR *ΔphoPR*::*hyg*, 10^3.6^ for *ΔphoPR*::*hyg*::*phoPR-H37Rv and* 10^3.5^ for *ΔphoPR*::*hyg*::*phoPR-STB-K)* and survival of mice was monitored. Endpoint was defined as loss of more than 20% of the bodyweight. Data are representative of two independent experiments. The difference between the experimental groups was evaluated by the log-rank (Mantel-Cox) test: P values, * P<0.05, ** P<0.01.

The results in macrophages suggest that the attenuation due to the deficient *phoPR*-allele in *M*. *canettii* STB-K is observed in absence of adaptive immune response. To confirm this, we infected severe combined immunodeficient (SCID) mice with 10^3^ cfu of each strain and monitored the time to humane endpoint (Fig 6). First, we confirmed the delay in the survival time between *MTB* H37Rv and *M*. *canettii* STB-KR (median survival time 25 days and 53 days for H37Rv and STB-KR respectively) previously reported [14]. We also showed a significant difference between Δ*phoPR*::*hyg* strains complemented with *phoPR-H37Rv* (50 days) and *phoPR-STB-K* (66 days). So, these results indicate that the Δ*phoPR*::*hyg* mutant expressing the *phoPR-STB-K* cause less mortality in SCID-mice than the isogenic strain expressing *phoPR-H37Rv*, which is consistent with the reduced intra-macrophagic replication of *phoPR-STB-K-*complemented strain.

Taken together, our results demonstrated that expression of *phoPR-H37Rv* in the genetic background of STB-KR enhanced the virulence both in immunocompetent and in immunodeficient mice.

### The PhoPR two-component systems from the various *M*. *canettii* strains display variable activity

**Fig 7:**
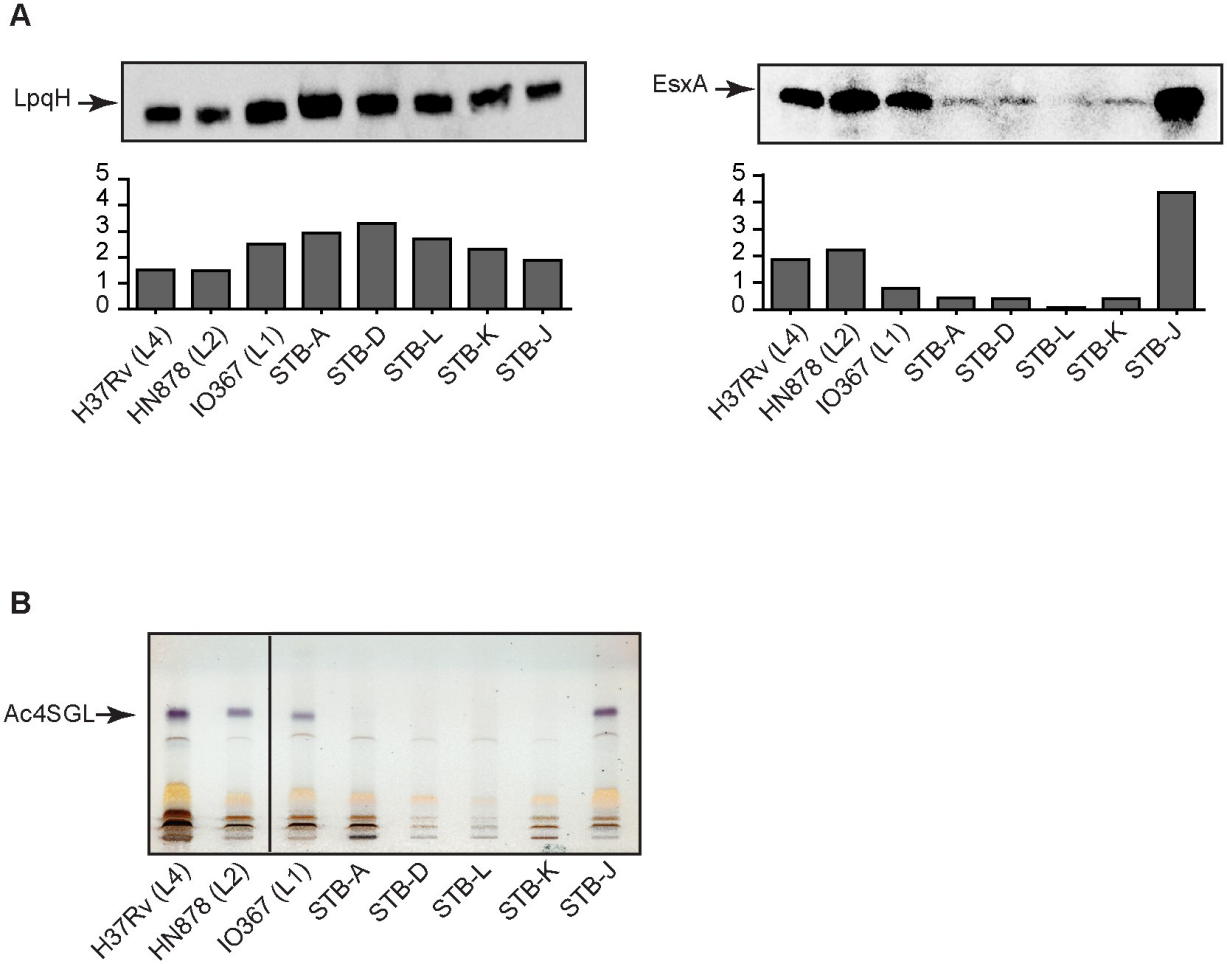
Most *M*. *canettii* isolates express PhoPR-regulated functions at lower level than *MTB* strains. A) Western blot analysis of proteins from the culture supernatants of MTBC and *M*. *canettii* strains. The protein extracts were probed with EsxA- or LpqH-specific antibodies. Signal intensity (arbitrary unit) was plotted in the graphs below the Western-blot images. The presented results are representative of two independent experiments. B) HP-TLC analysis of positively charged lipids extracted from various MTBC and *M*. *canettii* strains. Lipid extracts were dissolved in CHCl_3_ at a concentration of 20 mg/ml and 20 μl of extract were run in CHCl_3_/CH_3_OH/H_2_O, 60:16:2. Glycolipids were visualized by spraying with anthrone, followed by charring. The position of Ac4SGL is highlighted. The presented results are representative of two independent experiments.

The *phoPR* allele from STB-K is the most distantly related to that of H37Rv and is less active *in vitro* and *in vivo*, which, in consequence, is correlated with reduced virulence in human macrophages and mice. We next sought to extend these findings to *M*. *canettii* strains with *phoPR* alleles that are intermediate showing between 2 to 5 amino-acid substitutions in PhoR in comparison to that from H37Rv. We first evaluated the secretion of EsxA by five *M*. *canettii* and three *MTB* strains from three phylogenetic lineages. All tested strains secreted comparable amount of LpqH, used as a control (Fig 7A). In contrast, four out of five *M*. *canettii* strains, including *M*. *canetti* STB-K, were impaired for EsxA secretion in comparison to *MTB in vitro* (Fig 7A). The outlier among *M*. *canettii* was STB-J, which was found to secrete copious amount of EsxA. To confirm this, we also analyzed the production of the main form sulfoglycolipids (Ac4SGL), another function under the control of PhoPR in *MTB* [18,21]. As expected, the three *MTB* strains produced large amount of Ac4SGL whereas the *M*. *canettii* strains synthesized much lower quantities of these lipids, again with the exception of STB-J (Fig 7B).

**Fig 8:**
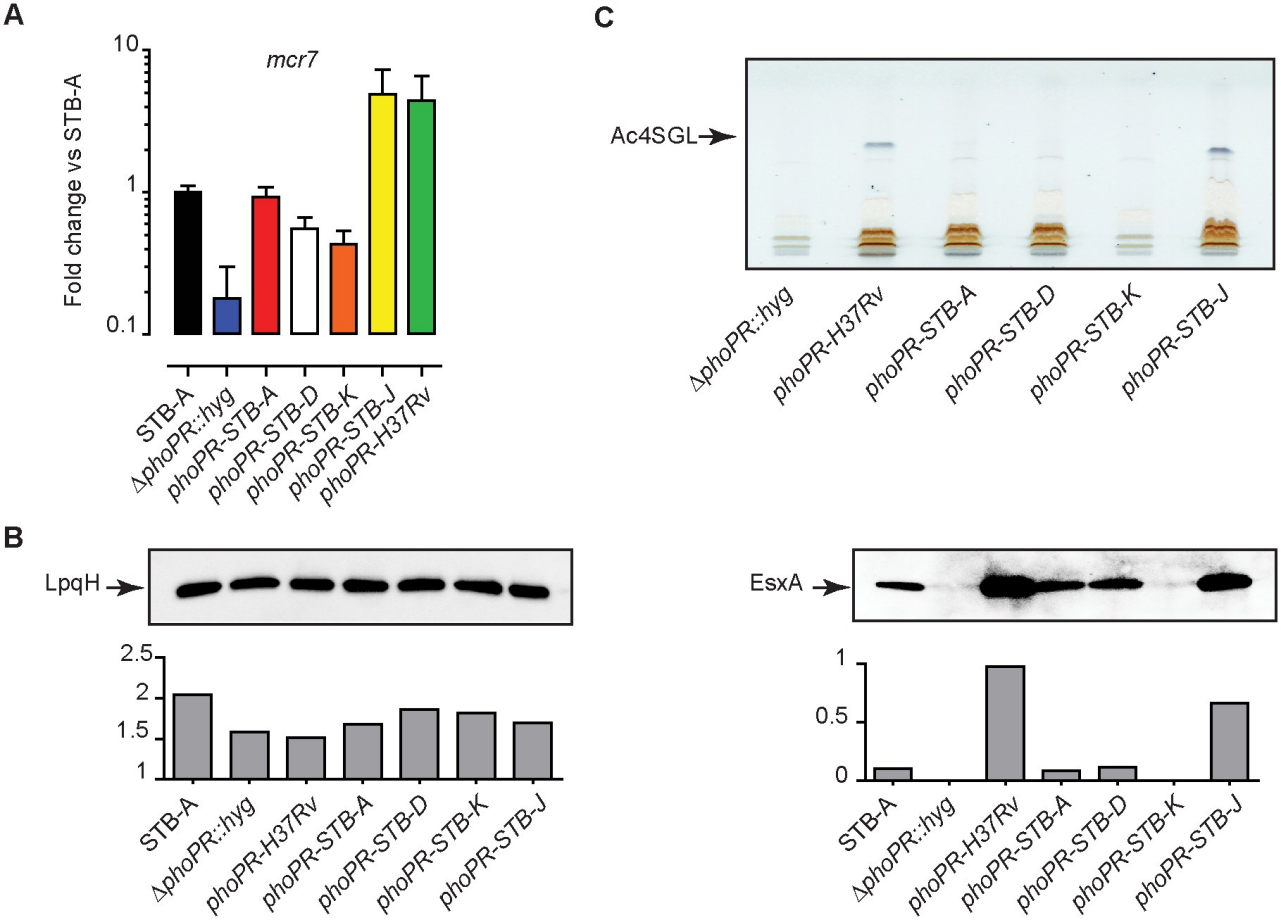
The various expression of PhoP-controlled function in *M*. *canettii* is linked to the *phoPR* alleles. A) RT-qPCR analysis of expression of *mcr7* in the *M*. *canettii* STB-A, *ΔphoPR*::*hyg* mutant or complemented strains expressing *phoPR* alleles from either H37Rv, STB-A, STB-D, STB-K, or STB-J. Bars represent fold changes in the *mcr7* expression levels relative to *M*.*canettii* STB-A wild-type. The presented results are means +/- SD of 3 independent experiments performed in triplicate. B) Immunoblot analysis of proteins from the culture supernatants of various strains derived from *M*. *canettii* STB-A *ΔphoPR*::*hyg* mutant. The protein extracts were probed with EsxA- or LpqH-specific antibodies. Signal intensity was plotted in the graph below the Western-blot image. The presented results are representative of two independent experiments. C) HP-TLC analysis of positively charged lipids extracted from *M*. *canettii* STB-A *ΔphoPR*::*hyg* mutant or complemented strains expressing *phoPR* alleles from either H37Rv, STB-A, STB-D, STB-K, or STB-J. Extracts enriched for positively charged lipids were dissolved in CHCl_3_ and run in CHCl_3_/CH_3_OH/H_2_O, 60:16:2, before spraying with anthrone and charring. The position of Ac4SGL is highlighted. The presented results are representative of two independent experiments.

To link these observations with the *phoPR* allele, we used a previously-constructed *phoPR*-knock-out mutant derived from STB-A, named STB-A Δ*phoPR*::*hyg* [36], into which 5 different *phoPR* alleles from STB-A, STB-D, STB-K, STB-J and H37Rv were integrated. We then compared the expression of *mcr7* in the parental STB-A strain, the STB-A Δ*phoPR*::*hyg* mutant and the 5 recombinant strains (Fig 8A). As expected, we found that complementation with *phoPR-STB-K* poorly induced the expression of *mcr7*. The recombinant strains carrying the *phoPR-STB-D* gave slightly higher level of *mcr7* expression and the complementation was almost complete with *phoPR-STB-A*. In contrast, the *phoPR-H37Rv* and *phoPR-STB-J* induced a robust transcription of *mcr7*, 5 and 6 fold above the STB-A WT control respectively. Analysis of EsxA secretion and sulfoglycolipids production confirmed the RT-qPCR results. Only expression of *phoPR-H37Rv* and *phoPR-STB-J* in STB-A Δ*phoPR*::*hyg* endowed the recombinant strains with the capacity to produce and secrete high amount of Ac4SGL and EsxA *in vitro* (Fig 8B and 8C). The *phoPR-STB-A* and *phoPR-STB-D* alleles gave EsxA secretion levels comparable to that of parental STB-A and only trace amount of Ac4SGL. Finally, complementation with *phoPR-STB-K* failed to induce production of detectable amount of Ac4SGL and secretion of substantial amounts of EsxA.

We next confirmed *in vivo* the various levels of EsxA secretion observed in the recombinant strains grown *in vitro*. The recombinant STB-A Δ*phoPR*::*hyg* strains were evaluated for their ability to induce EsxA-specific T-cell responses in mice (S12 Fig). We noted that, with the exception of the STB-A Δ*phoPR*::*hyg* mutant and the strain expressing *phoPR-STB-K*, all other strains were able to induce robust specific T-cell responses against EsxA but also EsxB and EspC (S12 Fig). These results are consistent with the *in vitro* observation showing that complementation of the STB-A Δ*phoPR*::*hyg* with *phoPR-STB-K* failed to restore secretion of substantial amount of EsxA.

In summary, our results established that there are strong differences in the expression of the PhoPR-regulon in *M*. *canettii* strains due to polymorphism of the *phoPR* genes. These differences were observed both *in vitro* and in infection models.

## Discussion

In this study, we established that several natural mutations found in the *phoR* gene from the various *M*. *canettii* strains have functional impact on the expression of the PhoP regulon both *in vitro* and *in vivo*. We identified three different behaviors of *phoPR* alleles from *M*. *canettii*. The *phoPR* from *M*. *canettii* STB-K, which displays the largest number of mutations in comparison to *phoPR-H37Rv*, is also the least active. In contrast, although the *phoPR-STB-J* is also very distant from *phoPR-H37Rv*, it provides the highest expression of PhoP-positively regulated genes. Between these two extremes, the *phoPR* alleles from STB-A and STB-D show intermediate expression of the PhoP-regulon. These differences were translated to PhoPR-regulated phenotypes such as EsxA secretion or sulfoglycolipid production.

Analysis of the amino acid sequence from PhoR-STB-K and PhoR-STB-J and the chemical environments of their specific mutations point toward two substitutions, respectively I268M and G430D, which may explain their unusual behavior. Residue Ile268, which occupies the same position with the same chemical environment in both the X-ray structure of the DHp domain and in the AlphaFold-multimer predicted structure, takes part to the 4-helix bundle that mediates dimerization and contains the phosphorylation site histidine. This residue is involved in both intrasprotomer (i.e. interactions between the two helices of a single protomer) and in dimer interface interactions, and contributes as such to the dimer hydrophobic core (S4C Fig) [26]. The mutation of the isoleucine at position 268 in PhoR-H37Rv to the methionine residue found in PhoR-STB-K, may well be accommodated during folding of the tertiary structure. In contrast, since residues 268 from the two protomers face each other (S4C Fig), the I268M substitution may cause steric conflicts, thereby affecting dimerization of the DHp domain. Since it has been proposed that the asymmetry found in the DHp crystal structure and its impact on the flexibility of PhoR is involved in the mechanism of the histidine kinase activities [26], the molecular rearrangement due to the I268M mutation at the PhoR dimer interface may indeed have functional consequences. In PhoR-STB-J, it is the G430D substitution, which likely explains the unusually high activity of this variant. Indeed, this change is the only amino acid substitution between PhoPR-STB-J and PhoR-STB-A, which does not exhibit the same high activity *in vitro* (Figs 7 and 8). The G430D substitution may stabilize the protein structure by creating interaction between aspartic residue 430 and two arginine residues at position 296 and 420 (S4J Fig). Residues 430 and 420 are found in the CA domain whereas residue 296 takes part to the DHp domain. Thus, in PhoR-STB-J, the interactions between Asp430 and Arg296 and Arg420 would both stabilize a badly predicted external loop of the CA domain and lock the CA domain to the nearby DHp domain.

Interestingly, the mutations found in *phoPR-STB-K* decrease the capacity of TB bacilli to replicate in infection models. In previous work, we already established that the smooth to rough morphotype transition in STB-K is associated with a higher capacity to replicate in human macrophages and in animal models [14]. Here, we found that expression of *phoPR-H37Rv* in STB-KR further enhances the virulence of the recombinant strain, although not to the level observed for *MTB* H37Rv. A recent study by Lovey and colleagues [37] has linked the capacity of *MTB* to transmit with the rapid induction of IL1R-dependent alveolar macrophage migration to the interstitium, a phenomenon associated with higher production of IL1β and TNFα in mice. Our observation that expression of *phoPR-H37Rv* in STB-K induces higher production of these cytokines in hMDMs than expression of *phoPR-STB-K* suggests that mutations in *phoR* may contribute to enhance the transmission capacity in TB bacilli.

Most of the other *M*. *canettii* strains tested, with the exception of STB-J, also display lower expression of PhoP-positively controlled functions than *MTB* strains *in vitro*. It is therefore tempting to speculate that adaptation of the phylogenetic branch of TB bacilli which evolved from an *M*. *canettii*-like progenitor into the clonal MTBC underwent mutations, including those seen today in *MTB*, to increase the expression of the PhoP-positively controlled genes, which made them better adapted to the mammalian host than this is the case for *M*. *canettii* strains. However, it is also clear that within the clonal MTBC, some lineages carry mutations in *phoR*, such as in *M*. *africanum* and animal-adapted MTBC members, that may reduce PhoPR-related activity and for which compensatory mutations, such as the deletion of the RD8 region, seem to have occurred [36]. Another example is the PhoR-H37Rv which carries a specific mutation at position aa. 172 (a proline rather than a leucine like in most PhoR from other *MTB* strains, such as CDC1551 or HN878) that seems to impacts the activity of the PhoPR system [25].

In summary, our study further underscores the crucial role played by PhoR in the continuous adaptation of TB bacilli to their host and suggests that emergence of *MTB* was accompanied by a selection of *phoPR* alleles favoring early replication within host cells. The identification of several mutations that modulate signal recognition/transduction and host/pathogen interaction in our study open fascinating perspectives to understand host/pathogen cross-talk in TB and adaptations that favor colonization and transmission in humans.

## Methods

### Ethics statement

Whole blood from donors was provided by Etablissement Français du Sang (EFS, Toulouse, France, under contract 21/PLER/TOU/IPBS01/2020-025). According to article L1243-57 of the French Public Health Code, the contract was declared to the French Ministry of Science and Technology (declaration number DC 2012–1715). Written informed consents were obtained from the donors before sample collection.

For virulence studies in mice, all protocols were reviewed and approved in compliance with the European Community council directive (EEC guidelines) and its implementation in France and received approval from the French Ministry for High Education and Research (number 201508271122464v2 (APAFIS#1535) and the Ethics Committee for Animal Experimentation from the Pasteur Institute (CETEA) # dap18002, (APAFIS#15409).

### Bacterial strains, plasmids and culture conditions

The strains and plasmids are described in S2 Table. These strains were cultured in Middlebrook 7H9 liquid medium (Difco) containing ADC (0.2% dextrose, 0.5% BSA fraction V, 0.0003% beef catalase) and 0.05% Tween 80 when indicated, and on solid Middlebrook 7H11 broth containing OADC (0.005% oleic acid, 0.2% dextrose, 0.5%BSA fraction V, 0.0003% beef catalase). When required, kanamycin (40μg/ml) or hygromycin (50μg/ml) were added to the culture medium.

The *M*. *canettii* STB-KR Δ*phoPR*::*hyg* was obtained using previously described strategy and primers [36]. The structure of the *phoPR* locus in the mutant strain was verified using PCR amplification with various primer couples (S6 Fig). For complementation, we obtained the various *phoPR-STB* alleles using the PCR amplification procedure and primers 0756c and 0759c described previously [36] and genomic DNA from each of the *M*. *canettii* strains. Each cloned fragments were sequenced to verify that no mutation was introduced during the amplification and DNA manipulation. The fragment covering the promotor and the *phoPR* genes were inserted within an integrative plasmid carrying a kanamycin resistance gene.

For experiments in human macrophages, *M*. *canettii* STB-KR WT and derivatives were made fluorescent by transferring a replicative plasmid carrying the GFP gene, pWM251 (S2 Table).

### RNAseq analysis

WT strains of *MTB* H37Rv and HN878, and *M*. *canettii* STB-KS and STB-KR were cultured in triplicate in 30 mL of Middlebrook 7H9 ADC Tween (0.05%) liquid medium at 37°C without agitation. Cultures were harvested when reaching the exponential phase of growth with a final OD_600nm_ between 0.5 and 0.8. RNA extraction was performed using a Trizol RNA isolation protocol (Invitrogen) and a mechanical lysis in Lysing Matrix B tubes (MP Biomedicals, Fisher Scientific) *via* rapid agitation in a Bead Mill 24 Homogenizer (Fisherbrand, Thermo Fisher Scientific). RNA extracts were then treated with the Turbo DNase (Ambion) and cleaned up using the RNeasy mini kit (Qiagen). RNA integrity and quality was monitored with a Bioanalyzer RNA nano assay (Agilent Technologies) to ensure that the RNA Integrity Number (RIN) was greater than 9. RNAseq libraries were prepared using the Stranded Total RNA Prep and Ligation with Ribo-Zero Plus kit (Illumina) and sequenced using a NextSeq 2000 device (Illumina). Generated strand-specific 100-bp single-end reads were mapped against the reference genome of *MTB* H37Rv (AL123456.3) [38] using BWA-MEM v0.7.17-r1188 [39] (parameters: -M; -h 1000). Uniquely-mapped reads were extracted from the alignment maps according to the XA tag using the Python wrapper pysam v0.20.0 (https://github.com/pysam-developers/pysam) of SAMtools [40]. Reads mapped on gene features were counted using featureCounts v2.0.4 [41] (parameters: -s 2;—primary). Counts associated with the genes *rrs*, *rrl* and *rrf*, encoding ribosomal RNAs, were excluded to prevent differences related to variable ribodepletion efficiencies during the library preparation of the samples. Read counts were normalized and transformed by regularized logarithm using DESeq2 v1.38.3 [42], and differential expression analysis was performed using a false-discovery rate (FDR, alpha) of 0.05. Genes with an absolute log2 fold change of at least 1 and an adjusted p-value (padj) lower than 0.05 were considered as differentially expressed (DE) (S1 Table). Lists of PhoP-regulated genes was taken from [18,23] and their expression level determined from the normalized and transformed read counts in the sequenced samples was plotted as heat maps using the R package ComplexHeatmap v2.14.0 [43]. Hierarchical clustering of the genes was performed using the complete-linkage method on Pearson correlation distances, while hierarchical clustering of the samples was performed using the complete-linkage method on Euclidean distances.

### RT-qPCR analyses

For RNA extraction, we grew 7ml culture of each strain in 7H9 ADC Tween 0.05% to a final OD_600nm_ between 0.5 and 0.8. The bacteria were pelleted by centrifugation 4000 rpm for 10 min and washed once with 1 ml PBS. The bacterial pellets were resuspended in 750 μl lysis buffer RLT (RNeasy kit, Qiagen) containing β-mercaptoethanol 0.1% (Sigma). Glass-beads were added to the bacteria before lysis by shaking twice in a bead-beater (2 cycles of 1 min). Bacterial lysates were filtered twice through 0.22 μm filters (13 mm PES filter, Sigma). The RNeasy kit was then used following supplier’s protocol. RNA were treated with DNaseI (Fisher scientific) before being retro-transcribed using Superscript III Reverse transcriptase (Invitrogen). RT-qPCR reaction were performed using the SensiFAST SYBR Hi-ROX kit according to supplier’s recommendation and specific primers (S3 Table). The gene *sigA* was used as housekeeping gene and the ΔΔCt method was used to calculate the relative gene expression.

### Western-Blot

For immunoblot analysis of secreted proteins, 1 ml of 3 weeks-old pre-culture was inoculated in 20 ml 7H9 supplemented with dextrose 0.2% and incubated for 10 days. Bacteria were pelleted at 3500 rpm for 15 min and supernatants were collected and filtered twice on PVDF 0.2 μm filters. Extracts were concentrated 50x using Amicon Ultra 3 kDa cartridge. Approximately 5 μg of proteins were separated on SDS/PAGE 4–20% gels (Mini-Protean TGX Precast Gel 10w) and transferred onto membranes. Primary antibodies (anti-EsxA (Abcam) and anti-LpqH (BEI resources)) and secondary HRP-conjugated Goat anti-mouse antibodies (BioRad) were used at dilution 1:1000 and 1:5000 respectively. Signals were revealed using Imobilon Western chemiluminescent subtstrate (Millipore) and ChemiDoc MP Imaging System (BioRad). Signal quantification was performed using Image Lab software (BioRad).

### Lipids analysis

Crude lipid extracts were obtained from 100 ml culture grown for 8 weeks in 7H9 ADC. Bacteria were recovered by centrifugation at 3500rpm for 15 min and lipids were extracted by adding 60 ml of CHCl_3_/CH_3_OH 1:2 for 48 h, followed by 30 ml of CHCl_3_/CH_3_OH 2:1 for 48 h. Organic phases were collected, pooled, concentrated, washed with water and evaporated to dryness. Approximately 10 mg of crude lipid extracts were obtained from each culture. For sulfoglycolipid purification, 5 mg of crude extract was applied to a Sillica Sep-Pak Accell Plus QMA Classic Cartridge 360 mg (Waters) and eluted by 10 ml CHCl_3_, 10 ml CHCl_3_/CH_3_OH 8:2, 10 ml CHCl_3_/CH_3_OH 5:5 and 2 ml CHCl_3_/CH_3_OH 5:5 containing 200 mM ammonium acetate. The last fraction, containing the sulfoglycolipids, was evaporated to dryness. Extracts were resuspended in 200 μl CHCl_3_ and analyzed on HPTLC (Camag) using CHCl_3_/CH_3_OH/H_2_O (60:16:2, vol/vol/vol). Glycolipids were visualized by spraying the plates with a 0.2% anthrone solution in concentrated H_2_SO_4_, followed by heating.

### Virulence studies in human macrophages

hMDMs were isolated as previously described [44] and cultured for 7 days on sterile glass coverslips in 24-well tissue culture plates (5.10^5^ cells/well) containing RPMI 1640 (Gibco) supplemented with 2 mM glutamine (Gibco) and 7% heat inactivated human AB serum. Infection of hMDMs and imaging of infected cells was performed as previously described [45]. The experiments were performed at least two times independently with cells from 4 or 5 independent donors and more than 100 cells from different fields were analyzed.

### Image analysis

Images from spinning disk (Andor Technology, Oxford Instruments compagny) acquisition were analyzed with macros (available upon request). All image analysis was performed using FIJI (https://imagej.net/Fiji) as previously described [45]. Briefly, to calculate the percentage of infected cells, images were split into their constitutive color channels and a z-projection summing the slices was used to visualize bacteria either bound at the surface of macrophages or ingested. We then enumerated the number of intracellular bacteria per cells and the percentage of macrophages having bound or ingested bacteria. To evaluate the intracellular bacterial load, we first delineate the location of individual cells by calculating the center of mass from the DAPI nuclei image and using the Voronoi network analysis to determine the region of interest (ROI) of individual cells. The ROI was applied to the green channel image and the sum of all GFP-positive pixels (RAWIntDen), corresponding to the GFP signal from the bacteria, was determined and quantified per infected cell. Values obtained for each lot of hMDMs were normalized by subtracting the mean fluorescence intensity value observed with H37Rv control for each experiment and by adding the mean fluorescence intensity value observed for all experiments with H37Rv.

### Cytokines production

For cytokine analysis, hMDMs were infected with various bacterial strains at MOI 2 for 2 h. At 48 h post infection, cell culture supernatants were removed, 0.22 μM filtered twice and processed for ELISA for IL1β, IL6, IL10 and TNFα according to the manufacturer’s instruction (Duoset R&D Systems, Lille, France). Data were expressed as picograms of cytokine per milliliter of cell culture medium.

### Virulence studies in mice

C3HeB/FeJ mice, 7 to 8 weeks old, were infected intravenously with approximately 10^3^ cfu per mouse. For time to death experiments, female SCID mice 7 week old (Janvier) were infected intranasally with approximately 10^3^ cfu of the indicated strains (five mice per group). Mice were euthanized when weight loss reached 20% of their body-weight. For histomorphological analyses, C3HeB/FeJ mice were euthanized by intraperitoneal injection of Dolethal at 10 weeks post-infection and lungs processed and fixed for 48 h in 10% neutral buffer formalin before transfer into 70% ethanol. After the lungs were embedded in paraffin, tissue samples were sectioned (3 μM) and stained with hematoxylin and eosin. Histomorphological scoring of TB lesions was determined by measuring area of infiltrated lung tissue using the Panoramic viewer software (3DHistech).

### Immunization, T-cell assay and ELISA

Eight-week-old C57BL/6JRj (H-2^b^) female mice (Janvier) were subcutaneously immunized at the base of the tail with ≈ 5 x 10^5^ cfu/mouse of different mycobacterial mutant strains. Four weeks post-immunization, splenocytes from the immunized mice (*n* = 2 mice/group) were pooled and cultured in 96-well plate (TPP) at 1 x 10^6^ cells/well in HL-1 medium (Biowhittaker) complemented with 2 mM GlutaMax (Invitrogen), 100 U/ml penicillin and 100 μg/ml streptomycin (Sigma) in presence of different mycobacterial ESX-1 and ESX-5 antigens as described in Sayes et al., 2012 [46]. Concanavalin A (2 μg/ml), Purified Protein Derivative (PPD, 5 μg/ml) and MHC-II-restricted Ag85A:241–260 peptide (5 μg/ml) were used as positive controls while rMalE protein, MalE:100–114 peptide and medium alone were used as negative controls. After 72 h of incubation at 37°C and 5% CO2, the IFN-γ production was quantified in the culture supernatants by ELISA. Nunc 96-well Maxisorp plate (Thermo-Fisher) were used and mAbs specific to IFN-γ (clone AN-18 for coating and clone R4-6A2 for detection, BD Pharmingen).

### Statistical analysis

Data were analyzed using the PRISM GraphPad software and statistical significance between experimental groups was determined either by one-way ANOVA followed Bonferroni’s comparison test for bacterial loads and lesions in mice and for percentage of hMDMs infected and cytokine analysis, or by log-rank (Mantel-Cox) test for time-to-death experiments. P values less than 0.05 were considered significant.

For statistical analysis of fluorescence intensity distribution after hMDM infection, Log10-transformed values were used to fit linear mixed models, where strains were implemented as fixed effect and donors as random intercepts. Models were fitted either for 2 h or 168 h data. Tukey post-hoc tests were carried out to identify significant comparisons between strains, for which adjusted p-values below 0.05 were considered as significant. Statistical analysis and visualization were carried out in R working environment [47] using the RStudio interface [48] and the following packages: lme4 [49], tidyverse [50], multcomp [51] and broom [52].

## Supporting information

S1 Text Analysis of the chemical environments of amino-acids differing in PhoR from H37Rv and variants from *M*. *canettii* strains. (DOCX)

S1 Fig Phylogenetic tree showing the distribution of selected *M*. *canettii* and *M*. *tuberculosis* complex (MTBC) strains. This phylogenetic tree is based on multilocus sequence typing results, using split decomposition analysis of concatenated sequence of 12 housekeeping gene segments. The scale bar represents Hamming distance, Figure adapted from [6]. *M*. *canettii* strains that were included in the current work are shown in bold.
(TIF)

S2 Fig Two artificial intelligence algorithms give similar predicted 3D structures of *MTB* PhoR. The results of the AlphaFold (colored cartoon) and RoseTTaFold (grey cartoon) predictions are shown. A) Overall superimposition. B) Per-domain superimposition. Putative domain boundaries and rmsd values/number of superimposed Cα atoms are given. For the superimposition of the CA domains, the last sixteen C-terminal residues, predicted with low confidence, were not taken into account. Color code: sensor loop (SL), blue; transmembrane helices (TMD), brown; signal-transducing domain (HAMP), orange; dimerization and histidine phosphotransfer (DHp), green; catalytic/ATP-binding domain (CA), violet. Regions of low confidence (pLDDT < 70) are in red. Putative boundaries of the domains are given. Figure generated using PyMOL (PyMOL Molecular Graphics System, Version 2.4.1 Schrödinger, LLC).
(TIF)

S3 Fig The predicted 3D structure of the PhoR DHp domain is highly similar to the determined X-ray structure. A) Ribbon representation of the predicted model from AlphaFold-Multimer (green) and X-ray structure (black ribbon) of the DHp domain (PD 5UKY). Mostly affected regions, i.e. displacement greater than rmsd after superimposition of Cα carbon atoms, are depicted in red on the X-ray structure. Figure generated using PyMOL. B) Displacement analysis after superimposition shown in A). Continuous and dashed lines are respectively for chain A and chain B, the two molecules making up the dimer. Red horizontal lines indicate 1 × rmsd.
(TIF)

S4 Fig Structure analysis of the different mutated positions in *M*. *canettii* PhoR. A) T35. B) P172. C) I268. D) S292. E) E317. F) C319. G) R335. H) T344. I) I375. J) G430. Each mutated position is shown as cyan sticks on the predicted model from AlphaFold-Multimer. Surrounding residues are shown as colored cartoon and lines (side chain) while residues found within 5 Å of the mutated positions are shown as enlarged sticks. The color code is the same as in Figs 1B and S2. Figure generated using PyMOL.
(TIF)

S5 Fig Expression levels of the genes regulated by PhoP in two *MTB* strains and two M. *canettii* STB-K isolates. Depicted genes correspond to the 44 positively-regulated (A) and the 70 negatively-regulated (B) by PhoP identified by Walters et al. [18]. Gene expression levels were calculated following normalization and regularized logarithm transformation of raw read counts determined by RNAseq.
(TIF)

S6 Fig Construction of the *M*. *canettii* STB-KR Δ*phoPR*::*hyg* mutant. A) PCR analyses of the *M*. *canettii* STB-KR Δ*phoPR*::*hyg*. The sequence of the various primers used is indicated. B) Schematic description of the genomic locus in *M*. *canettii* STB-KR and Δ*phoPR*::*hyg* mutant and of the strategy used to analyze the mutant.
(TIF)

S7 Fig Expression of *phoP* and *phoR* in the *M*. *canettii* STB-KR wild-type or recombinant strains. RT-qPCR analysis of expression of *phoP* (A) or *phoR* (B) genes in the *M*. *canettii* STB-KR, Δ*phoPR*::*hyg* mutant, and *phoPR-H37Rv* or *phoPR-STB-K* complemented strains. Bars represent fold changes in the expression levels of two genes relative to *M*. *canettii* STB-KR wild-type. The presented results are means +/- SD of 3 independent experiments performed in triplicate.
(TIF)

S8 Fig Expression of the ncRNA *mcr7* in the *M*. *canettii* STB-KR wild-type or recombinant strains grown under acidic conditions. For analysis of *mcr7* expression under acidic condition, the *M*. *canettii* STB-KR wild-type, Δ*phoPR*::*hyg*, and *phoPR-H37Rv or phoPR-STB-K* complemented strains were cultured for 4 days in 7H9 ADC Tween pH = 5.7. Bars represent fold changes in the expression levels of *mcr7* relative to *M*. *canettii* STB-KR wild-type. The presented results are means +/- SD of 3 independent experiments performed in triplicate.
(TIF)

S9 Fig *M*. *canettii* STB-KR does not induce EsxA-, EscpC- and EsxB-specific T cell responses in mice. T-cell IFN-γ responses in the spleen of C57BL/6 mice (*n* = 2 per group) subcutaneously immunized with 5 x 10^5^ cfu/mouse of different *MTB* or *M*. *canettii* mutant strains. Four weeks post-immunization, total splenocytes of the immunized mice were pooled and stimulated *ex vivo* with various ESX-1 (EsxA, EsxB and EspC) and ESX-5 (PE/PPE) secreted antigens during 72 h at 37°C. The IFN-γ was quantified in the culture supernatant by ELISA. Positive controls (ConA and PPD, black bars) and negative (rMalE, Ctrl peptide and medium alone, grey bars) controls were represented. Error bars represent SD.
(TIF)

S10 Fig Expression of *phoPR-H37Rv* in *M*. *canettii* STB-KR enhances infectivity in hMDMs. hMDMs were infected at a MOI of 2 bacteria per cell for 2 h with the various strains. After infection, cells were washed and further incubated with culture medium in the presence of serum. At 2 h and 168 h post-infection, cell nuclei and F-actin were labelled with DAPI and with rhodamine-phalloidin antibodies respectively. Bacteria were detected thanks to the GFP fluorescence. For each lot of hMDMs (derived from 5 different donors), the percentage of infected hMDMs and the fluorescence intensity per cell were evaluated. A) Vertical bar plots indicating the percentage of hMDMs hosting at least one bacterium after 2 h or 168 h of infection. The difference between the experimental groups was evaluated by one-way ANOVA and Bonferroni’s comparison test values. B) Box plot of the Log fluorescence intensity (RAWIntDen) measured for each strain at 2 h post-infection. For each strain, the mean value was also calculated and is indicated above the graph. C) Log fluorescence intensity (RAWIntDen) values plotted for each hMDM from each donor and each strain at 2 h post-infection. The difference between the experimental groups was evaluated as not significant using a mixed linear model. D) Cluster analysis of data plotted on panel C). E) Log fluorescence intensity (RAWIntDen) values plotted for each hMDM from each donor and each strain at 168 h post-infection. F) Cluster analysis of data plotted on panel E).
(TIF)

S11 Fig Lungs of C3HeB/FeJ mice infected with *M*. *canettii* STB-KR expressing *phoPR-H37Rv* tends to display higher number of lung lesions than the isogenic strain expressing *phoPR-STB-K*. Mice were infected with approximately 10^3^ cfu (10^3.9^ for STB-KR, 10^3.9^ for H37Rv, 10^3.3^ for STB-KR *ΔphoPR*::*hyg*, 10^3.7^ for *ΔphoPR*::*hyg*::*phoPR-H37Rv and* 10^3.6^ for *ΔphoPR*::*hyg*::*phoPR-STB-K)*. Lungs were recovered 70 days post-infection and processed for histopathology analysis. Mid-lung sections were stained with hematoxylin and eosin and analyzed. The number of identified lesions is plotted. The difference between the experimental groups was evaluated by one-way ANOVA and Bonferroni’s comparison test. P values, * P<0.05, ** P<0.01.
(TIF)

S12 Fig Only *M*. *canettii* STB-A expressing *phoPR-STB-K* does not induce EsxA-, EspC- and EsxB-specific T cells in mice. T-cell immune responses of C57BL/6 mice (*n* = 2 per group) s.c. immunized with 5 x 10^5^ CFU/mouse of different *M*. *canettii* mutant or complemented strains. Pool of splenocytes of the immunized mice were cultured *ex vivo* with various ESX-1 (EsxA, EsxB and EspC) and ESX-5 (PE/PPE) associated antigens during 72 h at 37°C. The IFN-γ was quantified in the culture supernatant by ELISA. Positive controls (black bars) and negative controls (grey bars) were represented. Error bars represent SD.
(TIF)

S1 Table DESeq2 output table of the differential expression analysis between *MTB* HN878, *M*. *canettii* STB-KS and STB-KR vs. *MTB* H37Rv. (XLSX)

S2 Table Name and main features of strains, plasmids, and phages used in this study. References are listed in S1 Text.
(DOCX)

S3 Table Name and sequences of primers used in this study. (DOCX)

S4 Table. N umerical values used to generate graphs. (XLSX)
