# Supplementary material for: Natural mutations in the sensor kinase of the PhoPR two-component regulatory system modulate virulence of ancestor-like tuberculosis bacilli
Source: PLoS Pathog. 2023 Jul 14;19(7):e1011437. doi: 10.1371/journal.ppat.1011437 (PMC10348564; doi:10.1371/journal.ppat.1011437)
Supplement: S1 Text — (DOCX) [file ppat.1011437.s001.docx]

**Supplementary information**

**Natural mutations in the sensor kinase of the PhoPR two-component regulatory system modulate virulence of ancestor-like tuberculosis bacilli.**

Wladimir Malaga**^1^**^#^, Delphine Payros**^1^**^#^, Eva Meunier**^1^**^#^, Wafa Frigui^2^, Fadel Sayes^2^, Alexandre Pawlik^2^, Mickael Orgeur^2^, Céline Berrone**^1^**, Flavie Moreau**^1^**, Serge Mazères**^1^**, Jesus Gonzalo-Asensio^3,4^, David Rengel**^1^**, Carlos Martin^3,4,5^, Catherine Astarie-Dequeker**^1^,** Lionel Mourey**^1^*,** Roland Brosch^2^* & Christophe Guilhot**^1^***

**Short Title:** *phoPR* polymorphism in *Mycobacterium canettii*

**S1 Text**

**Analysis of the chemical environments of amino-acids differing in PhoR from H37Rv and variants from *M. canettii* strains.**

Although AlphaFold is able to predict wild-type 3D structures of proteins very efficiently and with high accuracy, it fails in its current state to predict the impact of missense mutations on aggregation, misfolding and dysfunction [[1](#_ENREF_1)]. Thus, in the following paragraphs we will only describe the positions of the PhoR mutations and their chemical environments (S4 Fig).

**T35S** (S4A Fig): Thr35 is at the junction between the TMD and SL domains in a 4-helix bundle formed between the two PhoR protomers. The Thr35 side chains from the two protomers points toward the exterior of the bundle and their OG atom is at hydrogen bond distance of the Gln161 side chain OE1 atom. This hydrogen bond should be maintained in T35S mutant and the effect of the Thr by Ser replacement cannot therefore be easily foreseen.

**P172L** (S4B Fig): Pro172 is found in the part of the 4-helix bundle that spans the membrane. It is in very close contact to Leu22 from the other protomer. Thus, its mutation in leucine could lead to steric conflicts, which would require rearrangements in the corresponding helices and in turn may induce functional effects.

**I268M** (S4C Fig): Ile268 takes part to the 4-helix bundle of the DHp domain in a region where the predicted model superimposed very well with the DHp crystal structure (S3 Fig). The isoleucine side chains from the two protomers face each other in a hydrophobic environment and their replacement by a methionine should also induce steric conflicts which may lead to functional consequences.

**S292G** (S4D Fig): Ser292 also takes part to the DHp domain in a well predicted region with respect to the DHp crystal structure (S3 Fig). In the two protomers, their side chains are pointing out towards the exterior and are accessible to the milieu. Their replacement by a glycine should be easily accommodated but would induce the loss of the hydrogen bond found between the hydroxyl oxygen atom of the serine and the main chain oxygen atom of the neighboring Ser288.

**E317Q** (S4E Fig): Glu317 is at the periphery of the CA domain and points towards the outside. Being freely accessible, it is not engaged in any interaction and its mutation in glutamine should have no effect. However, it is noteworthy that this part of the structure is capped by the C terminus of the protein which is badly predicted by AlphaFold.

**C319R** (S4F Fig): Cys319 is located two residues further away than Glu317 in a part of the CA domain that is highly accessible to the outside environment. The mutation might have to induce a rearrangement of the arginine side chain to avoid steric conflicts with that of the Glu360 located just opposite and with which it could form a stabilizing salt bridge.

**R335L** (S4G Fig): Arg335 is also found in the CA domain. Since it is solvent exposed and not involved in any interaction, its replacement by a leucine residue may be accommodated without any change from a structure-function point of view.

**T344S** (S4H Fig): Thr344 is in the same environment as Arg335 and also exposed to solvent. It makes a hydrogen bond with the facing Thr385. This hydrogen bond should be easily maintained upon replacement by a serine.

**I375M** (S4I Fig): Ile375 is found on the same face of the CA domain as Arg335 and Thr344. Its side chain is nicely fitted in a hydrophobic pocket whose entrance is partly exposed to the milieu. The mutation to a methionine should be easily accommodated.

**G430D** (S4J Fig): Gly430 sits in a badly predicted external loop of the CA domain at the interface with the DHp domain. Its mutation to an aspartic residue would not seem to disturb the structure. On the contrary, it could allow the creation of ionic interactions with one and/or the other of the two arginines (Arg 296 and Arg420) located opposite, hence a potential stabilizing effect.

**References:**

1. Buel GR, Walters KJ. Can AlphaFold2 predict the impact of missense mutations on structure? Nat Struct Mol Biol. 2022;29:1-2.

2. Supply P, Marceau M, Mangenot S, Roche D, Rouanet C, Khanna V, et al. Genomic analysis of smooth tubercle bacilli provides insights into ancestry and pathoadaptation of *Mycobacterium tuberculosis*. Nat Genet. 2013;45:172-9.

3. Walters SB, Dubnau E, Kolesnikova I, Laval F, Daffé M, Smith I. The *Mycobacterium tuberculosis* PhoPR two-component system regulates genes essential for virulence and complex lipid biosynthesis. Mol Microbiol. 2006;60:312-30.

4. Cole ST, Brosch R, Parkhill J, Garnier T, Churcher C, Harris D, et al. Deciphering the biology of *Mycobacterium tuberculosis* from the complete genome sequence. Nature. 1998;393:537-44.

5. Manca C, Tsenova L, Bergtold A, Freeman S, Tovey M, Musser JM, et al. Virulence of a *Mycobacterium tuberculosis* clinical isolate in mice is determined by failure to induce Th1 type immunity and is associated with induction of IFN-alpha/beta. Proc Natl Acad Sci USA. 2001;98:5752-7.

6. Boritsch EC, Frigui W, Cascioferro A, Malaga W, Etienne G, Laval F, et al. pks5-recombination-mediated surface remodelling in Mycobacterium tuberculosis emergence. Nat Microbiol. 2016;1:15019.

7. Gonzalo-Asensio J, Malaga W, Pawlik A, Astarie-Dequeker C, Passemar C, Moreau F, et al. Evolutionary history of tuberculosis shaped by conserved mutations in the PhoPR virulence regulator. Proc Natl Acad Sci USA. 2014;111:11491-6.

8. Payros D, Alonso H, Malaga W, Volle A, Mazeres S, Dejean S, et al. Rv0180c contributes to *Mycobacterium tuberculosis* cell shape and to infectivity in mice and macrophages. PLOS Pathogens. 2021;17:e1010020.
