# Supplementary figures and images for: Natural mutations in the sensor kinase of the PhoPR two-component regulatory system modulate virulence of ancestor-like tuberculosis bacilli
Source: PLoS Pathog. 2023 Jul 14;19(7):e1011437. doi: 10.1371/journal.ppat.1011437 (PMC10348564; doi:10.1371/journal.ppat.1011437)

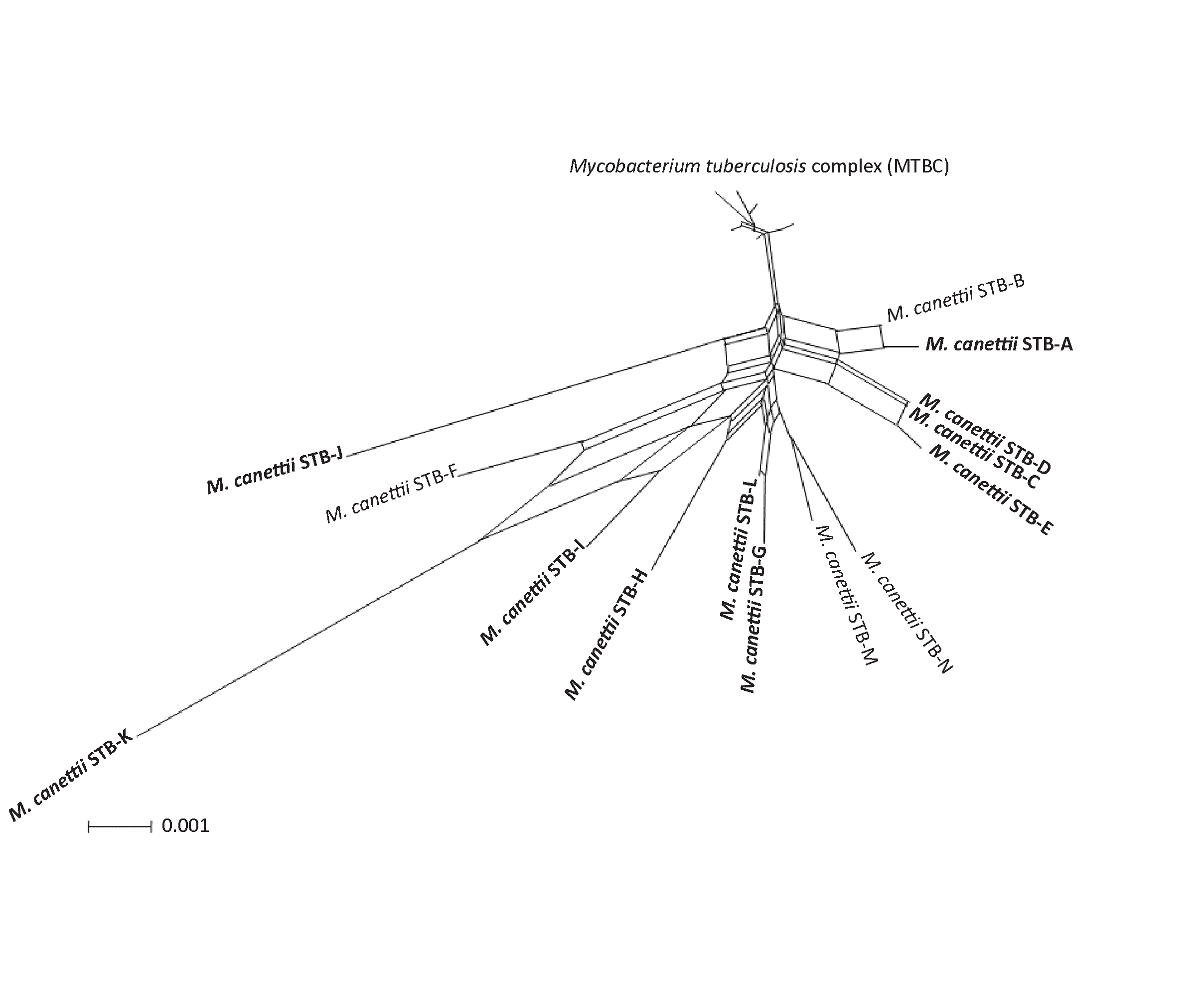

Supplement: S1 Fig — This phylogenetic tree is based on multilocus sequence typing results, using split decomposition analysis of concatenated sequence of 12 housekeeping gene segments. The scale bar represents Hamming distance, Figure adapted from [6]. M. canettii strains that were included in the current work are shown in bold. (TIF) [file ppat.1011437.s002.tif]

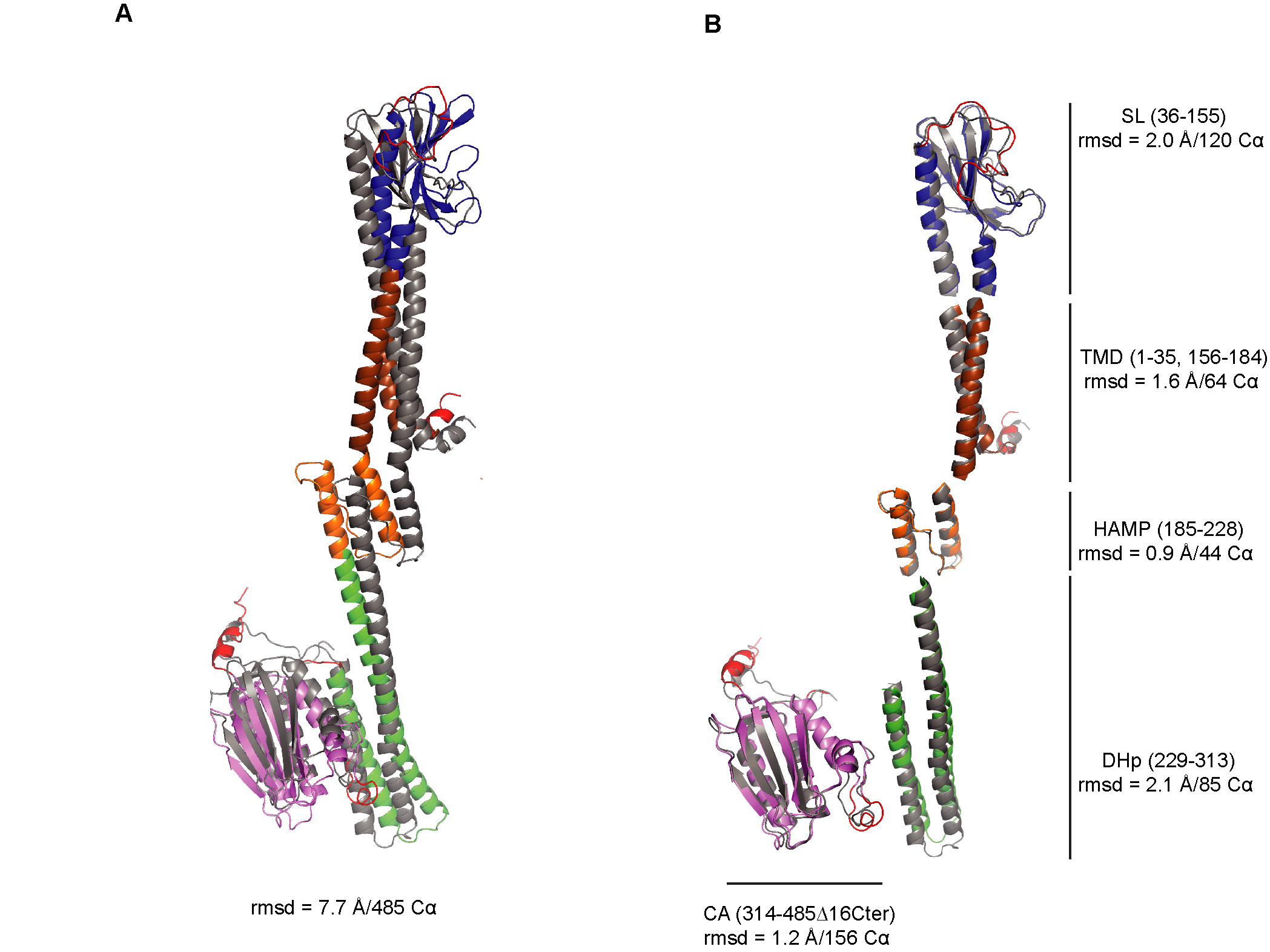

Supplement: S2 Fig — The results of the AlphaFold (colored cartoon) and RoseTTaFold (grey cartoon) predictions are shown. A) Overall superimposition. B) Per-domain superimposition. Putative domain boundaries and rmsd values/number of superimposed Cα atoms are given. For the superimposition of the CA domains, the last sixteen C-terminal residues, predicted with low confidence, were not taken into account. Color code: sensor loop (SL), blue; transmembrane helices (TMD), brown; signal-transducing domain (HAMP), orange; dimerization and histidine phosphotransfer (DHp), green; catalytic/ATP-binding domain (CA), violet. Regions of low confidence (pLDDT < 70) are in red. Putative boundaries of the domains are given. Figure generated using PyMOL (PyMOL Molecular Graphics System, Version 2.4.1 Schrödinger, LLC). (TIF) [file ppat.1011437.s003.tif]

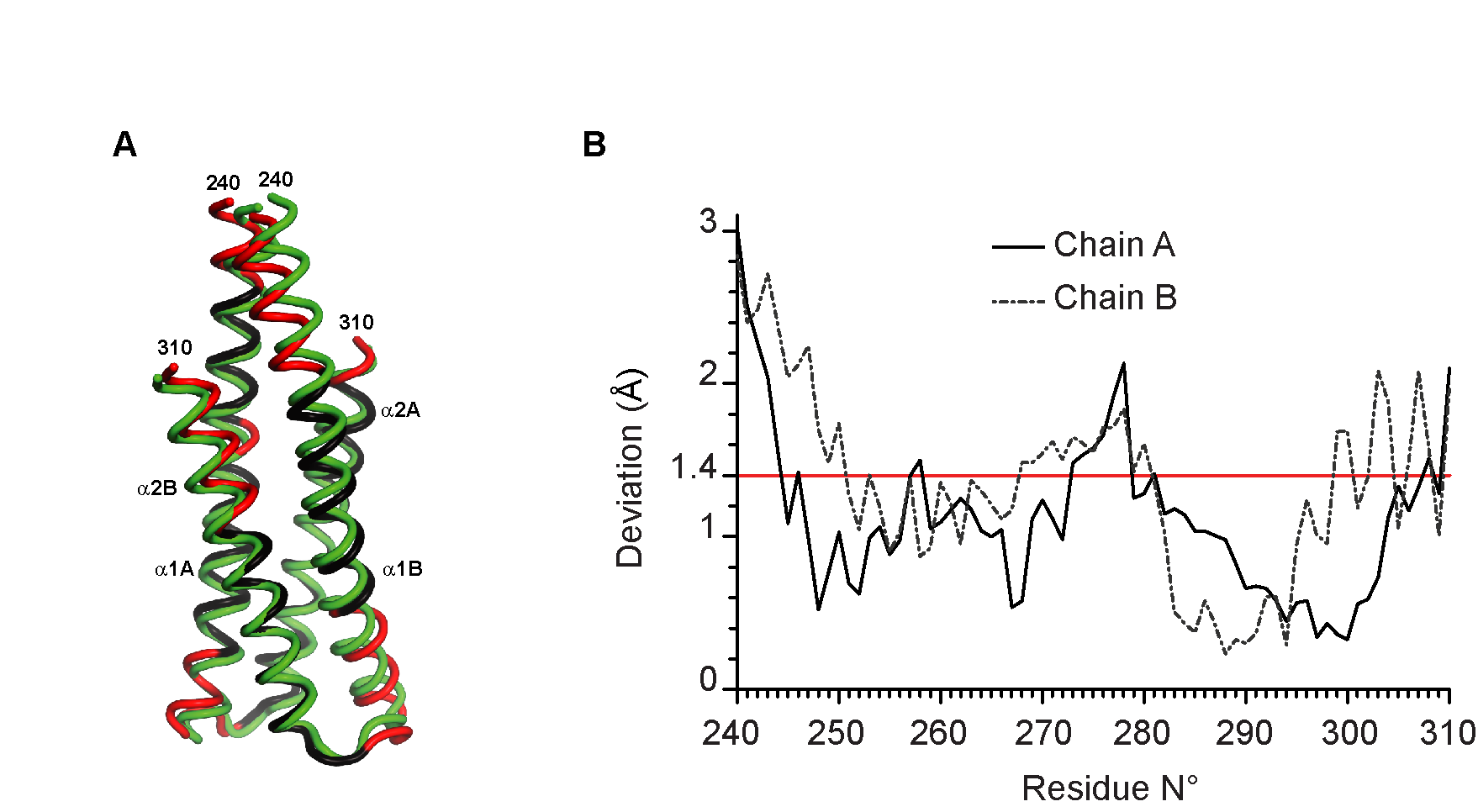

Supplement: S3 Fig — A) Ribbon representation of the predicted model from AlphaFold-Multimer (green) and X-ray structure (black ribbon) of the DHp domain (PD 5UKY). Mostly affected regions, i.e. displacement greater than rmsd after superimposition of Cα carbon atoms, are depicted in red on the X-ray structure. Figure generated using PyMOL. B) Displacement analysis after superimposition shown in A). Continuous and dashed lines are respectively for chain A and chain B, the two molecules making up the dimer. Red horizontal lines indicate 1 × rmsd. (TIF) [file ppat.1011437.s004.tif]

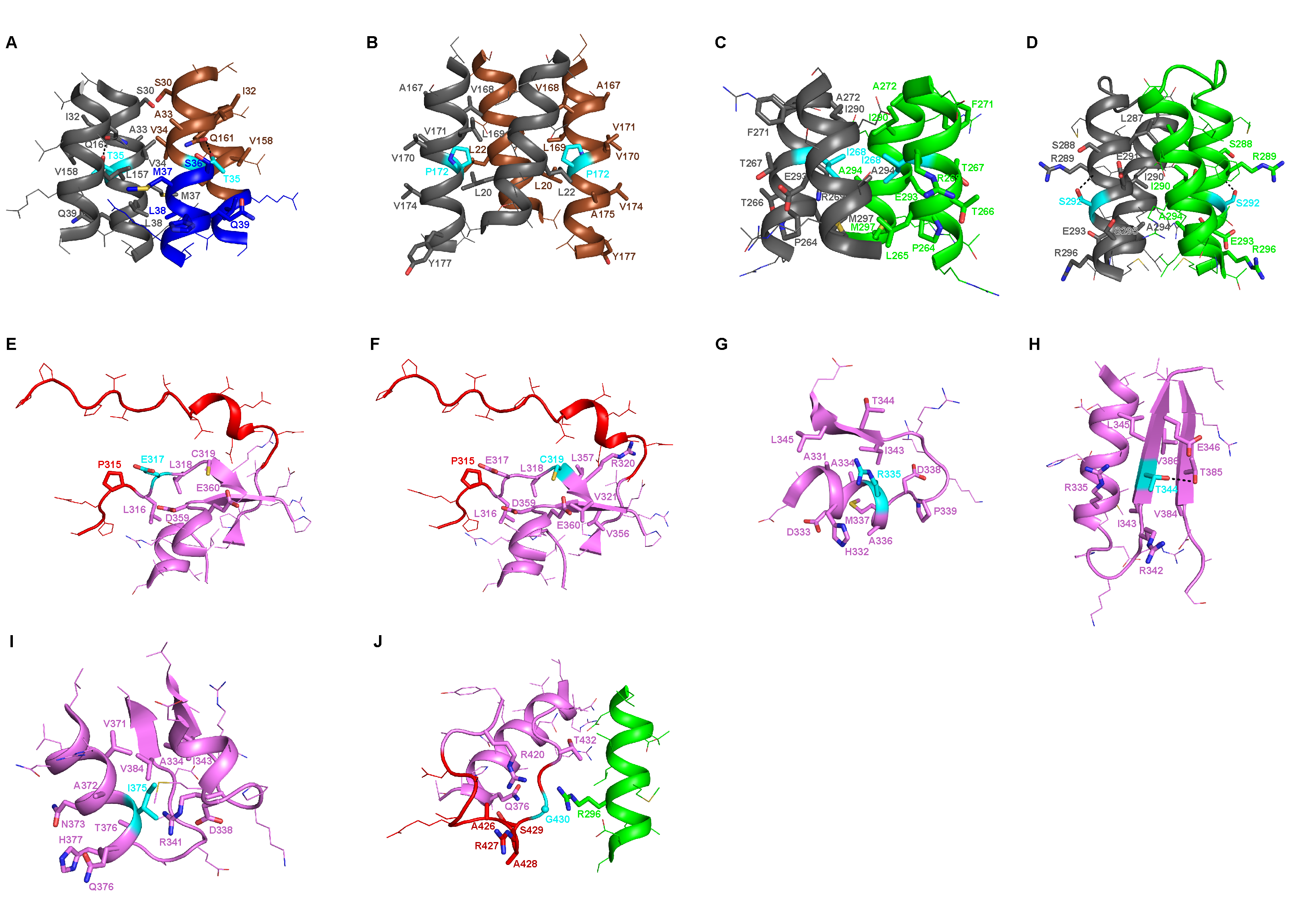

Supplement: S4 Fig — A) T35. B) P172. C) I268. D) S292. E) E317. F) C319. G) R335. H) T344. I) I375. J) G430. Each mutated position is shown as cyan sticks on the predicted model from AlphaFold-Multimer. Surrounding residues are shown as colored cartoon and lines (side chain) while residues found within 5 Å of the mutated positions are shown as enlarged sticks. The color code is the same as in Figs 1B and S2. Figure generated using PyMOL. (TIF) [file ppat.1011437.s005.tif]

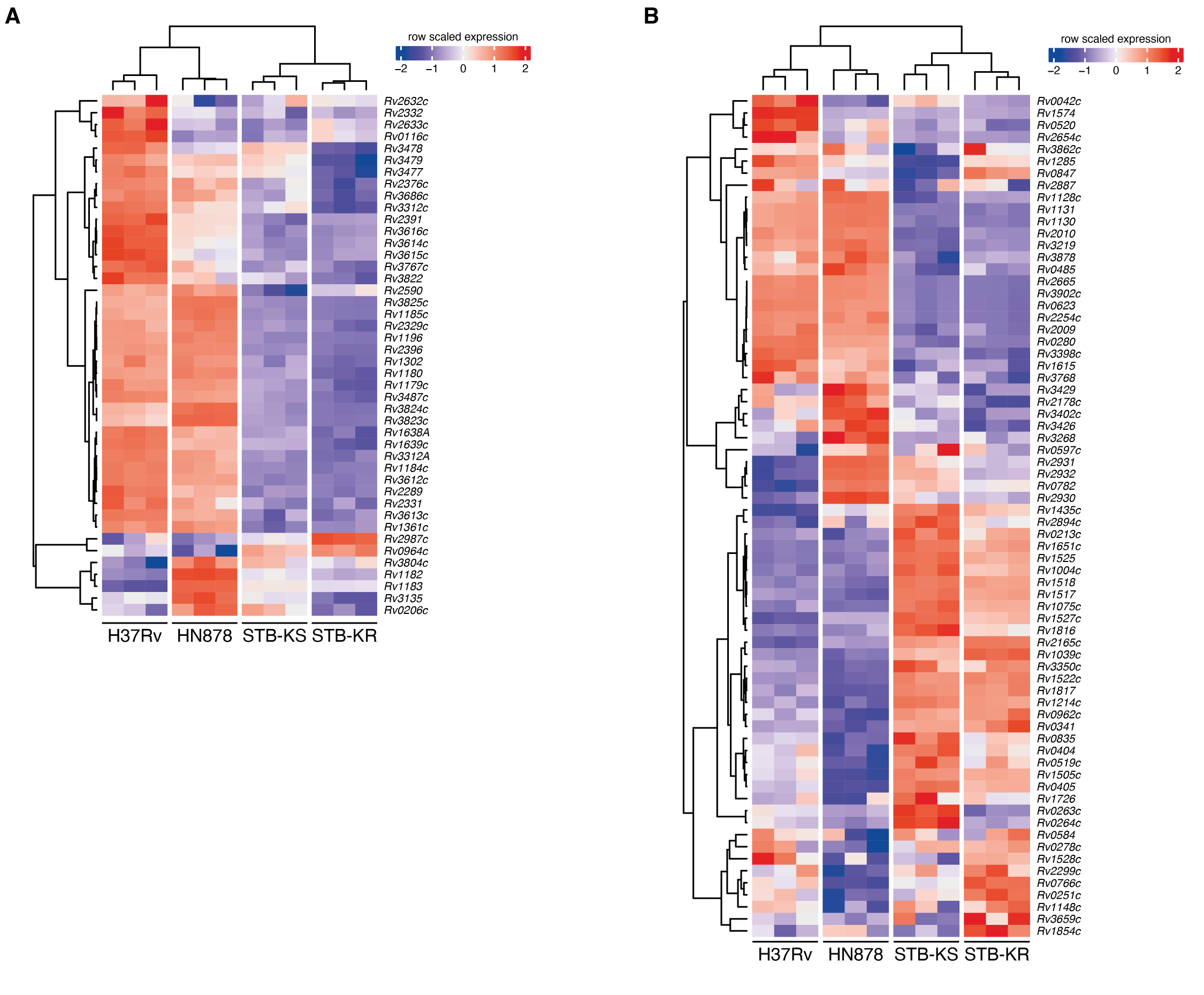

Supplement: S5 Fig — Depicted genes correspond to the 44 positively-regulated (A) and the 70 negatively-regulated (B) by PhoP identified by Walters et al. [18]. Gene expression levels were calculated following normalization and regularized logarithm transformation of raw read counts determined by RNAseq. (TIF) [file ppat.1011437.s006.tif]

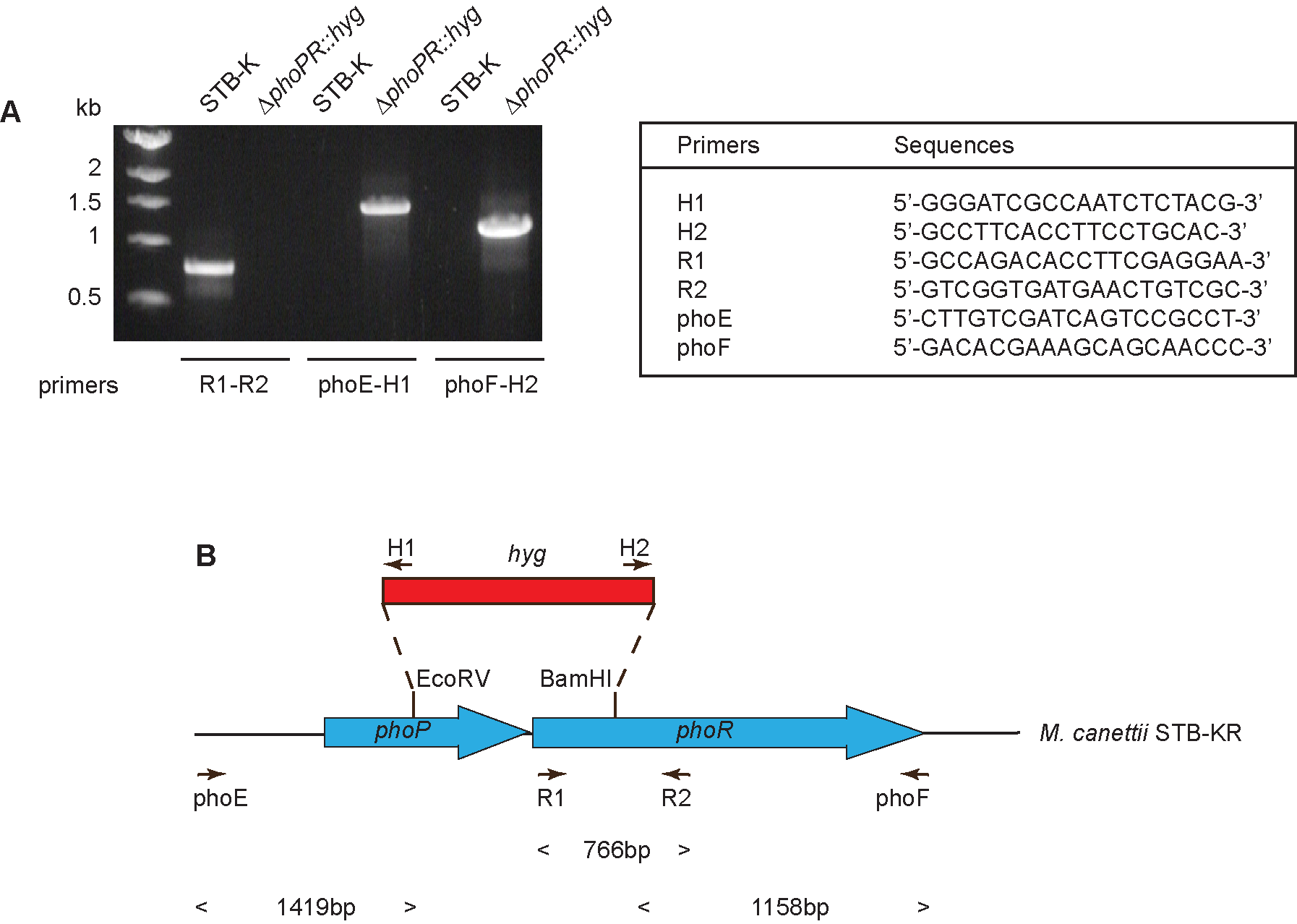

Supplement: S6 Fig — A) PCR analyses of the M. canettii STB-KR ΔphoPR::hyg. The sequence of the various primers used is indicated. B) Schematic description of the genomic locus in M. canettii STB-KR and ΔphoPR::hyg mutant and of the strategy used to analyze the mutant. (TIF) [file ppat.1011437.s007.tif]

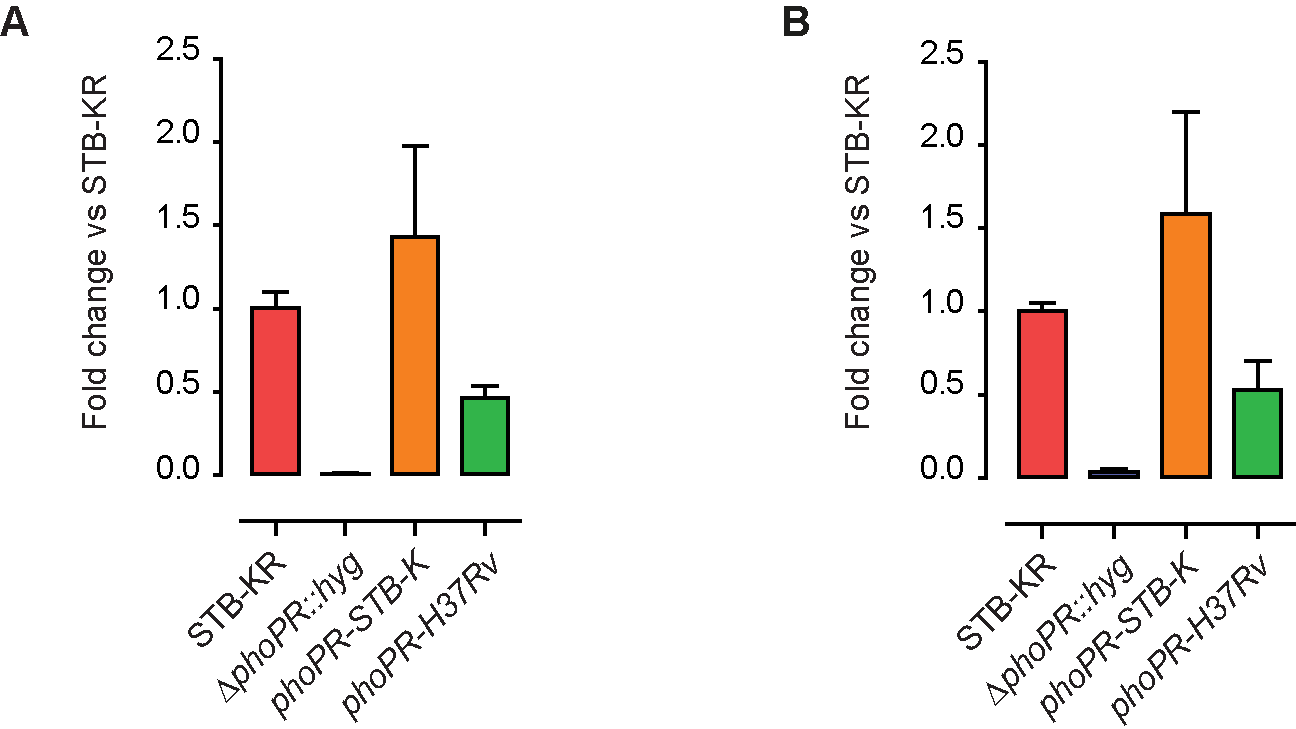

Supplement: S7 Fig — RT-qPCR analysis of expression of phoP (A) or phoR (B) genes in the M. canettii STB-KR, ΔphoPR::hyg mutant, and phoPR-H37Rv or phoPR-STB-K complemented strains. Bars represent fold changes in the expression levels of two genes relative to M. canettii STB-KR wild-type. The presented results are means +/- SD of 3 independent experiments performed in triplicate. (TIF) [file ppat.1011437.s008.tif]

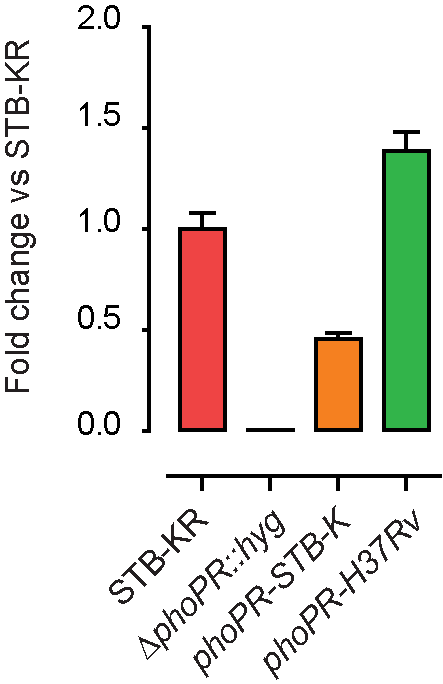

Supplement: S8 Fig — For analysis of mcr7 expression under acidic condition, the M. canettii STB-KR wild-type, ΔphoPR::hyg, and phoPR-H37Rv or phoPR-STB-K complemented strains were cultured for 4 days in 7H9 ADC Tween pH = 5.7. Bars represent fold changes in the expression levels of mcr7 relative to M. canettii STB-KR wild-type. The presented results are means +/- SD of 3 independent experiments performed in triplicate. (TIF) [file ppat.1011437.s009.tif]

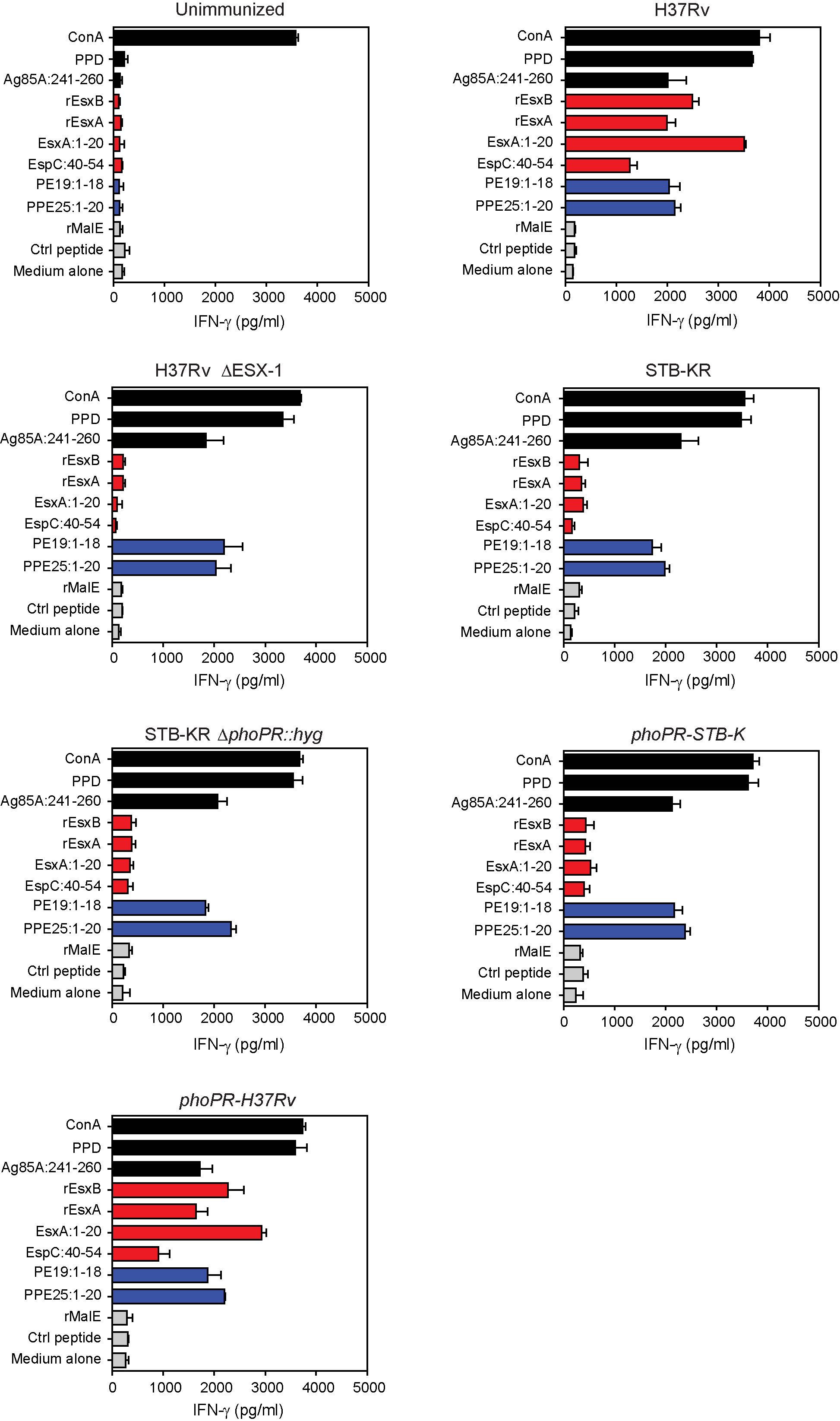

Supplement: S9 Fig — T-cell IFN-γ responses in the spleen of C57BL/6 mice (n = 2 per group) subcutaneously immunized with 5 x 10^5^ cfu/mouse of different MTB or M. canettii mutant strains. Four weeks post-immunization, total splenocytes of the immunized mice were pooled and stimulated ex vivo with various ESX-1 (EsxA, EsxB and EspC) and ESX-5 (PE/PPE) secreted antigens during 72 h at 37°C. The IFN-γ was quantified in the culture supernatant by ELISA. Positive controls (ConA and PPD, black bars) and negative (rMalE, Ctrl peptide and medium alone, grey bars) controls were represented. Error bars represent SD. (TIF) [file ppat.1011437.s010.tif]

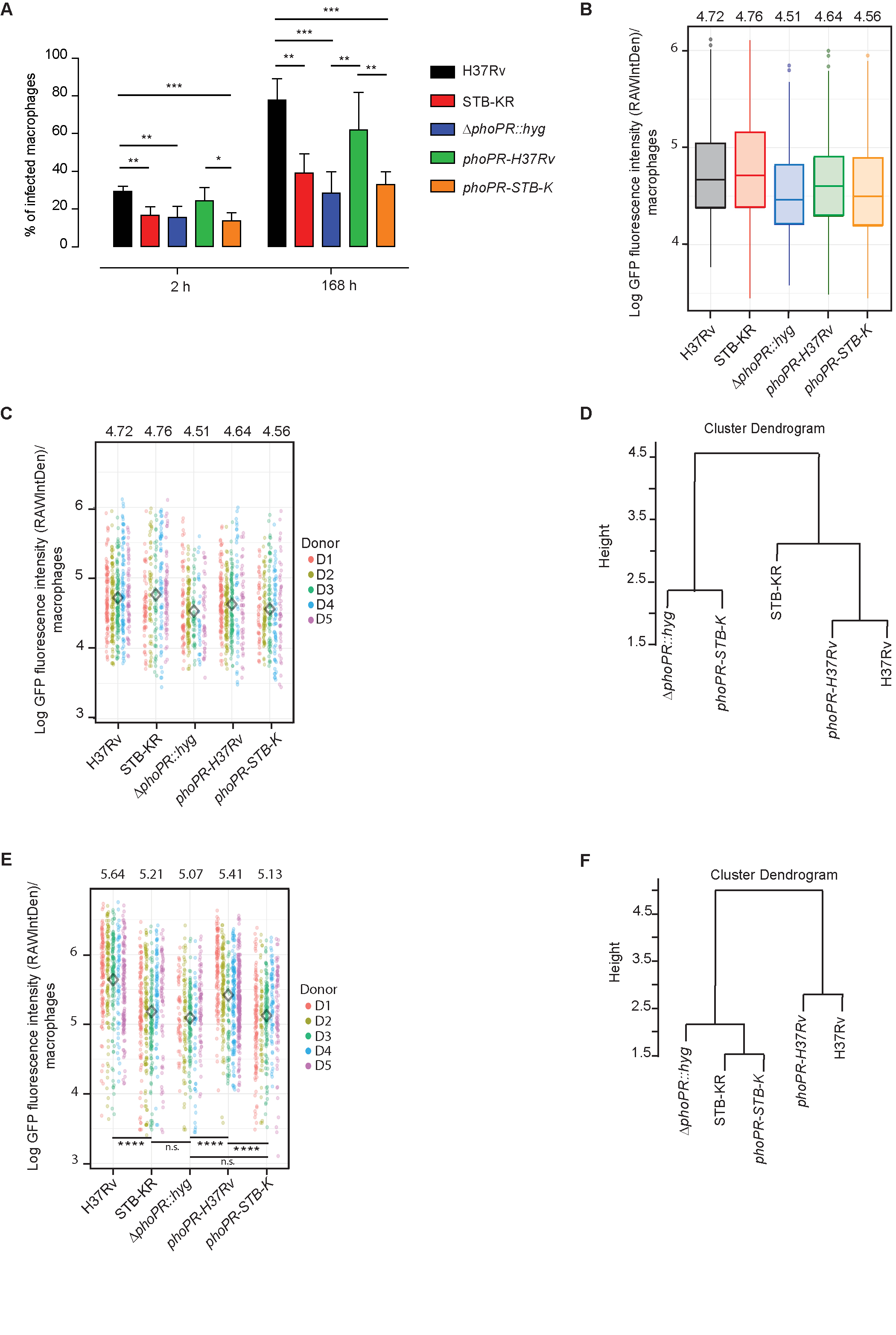

Supplement: S10 Fig — hMDMs were infected at a MOI of 2 bacteria per cell for 2 h with the various strains. After infection, cells were washed and further incubated with culture medium in the presence of serum. At 2 h and 168 h post-infection, cell nuclei and F-actin were labelled with DAPI and with rhodamine-phalloidin antibodies respectively. Bacteria were detected thanks to the GFP fluorescence. For each lot of hMDMs (derived from 5 different donors), the percentage of infected hMDMs and the fluorescence intensity per cell were evaluated. A) Vertical bar plots indicating the percentage of hMDMs hosting at least one bacterium after 2 h or 168 h of infection. The difference between the experimental groups was evaluated by one-way ANOVA and Bonferroni’s comparison test values. B) Box plot of the Log fluorescence intensity (RAWIntDen) measured for each strain at 2 h post-infection. For each strain, the mean value was also calculated and is indicated above the graph. C) Log fluorescence intensity (RAWIntDen) values plotted for each hMDM from each donor and each strain at 2 h post-infection. The difference between the experimental groups was evaluated as not significant using a mixed linear model. D) Cluster analysis of data plotted on panel C). E) Log fluorescence intensity (RAWIntDen) values plotted for each hMDM from each donor and each strain at 168 h post-infection. F) Cluster analysis of data plotted on panel E). (TIF) [file ppat.1011437.s011.tif]

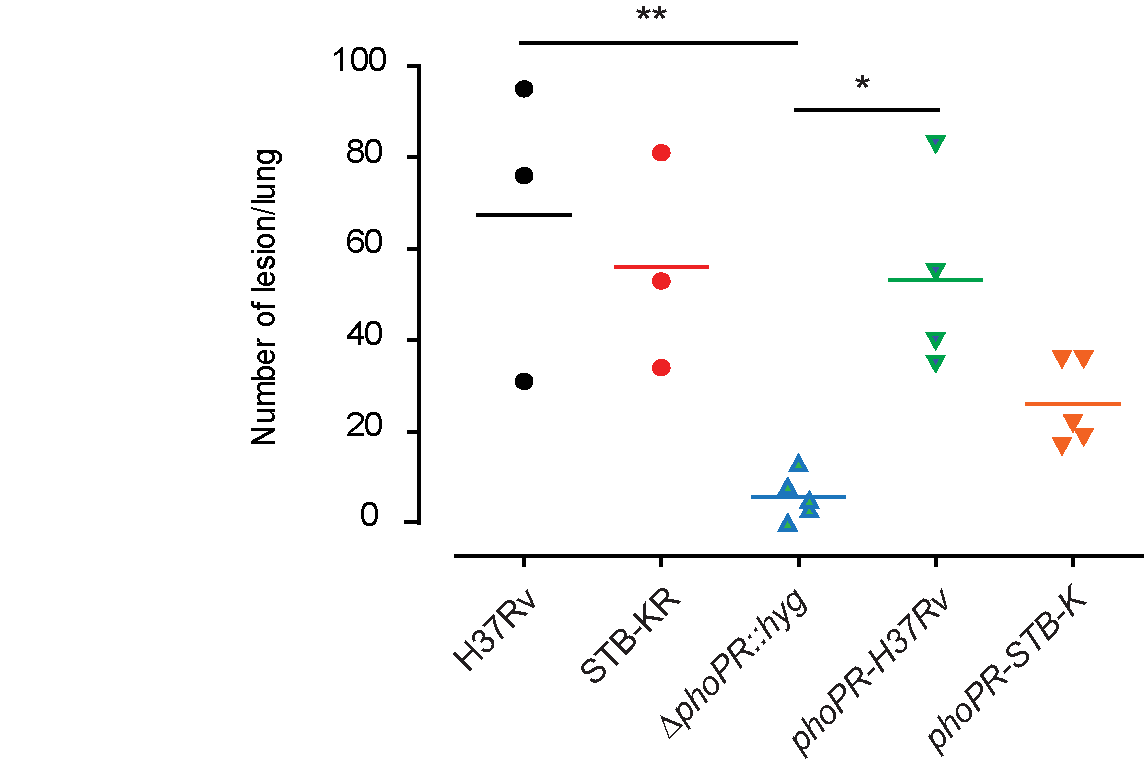

Supplement: S11 Fig — Mice were infected with approximately 103 cfu (103.9 for STB-KR, 103.9 for H37Rv, 103.3 for STB-KR ΔphoPR::hyg, 103.7 for ΔphoPR::hyg::phoPR-H37Rv and 103.6 for ΔphoPR::hyg::phoPR-STB-K). Lungs were recovered 70 days post-infection and processed for histopathology analysis. Mid-lung sections were stained with hematoxylin and eosin and analyzed. The number of identified lesions is plotted. The difference between the experimental groups was evaluated by one-way ANOVA and Bonferroni’s comparison test. P values, * P<0.05, ** P<0.01. (TIF) [file ppat.1011437.s012.tif]

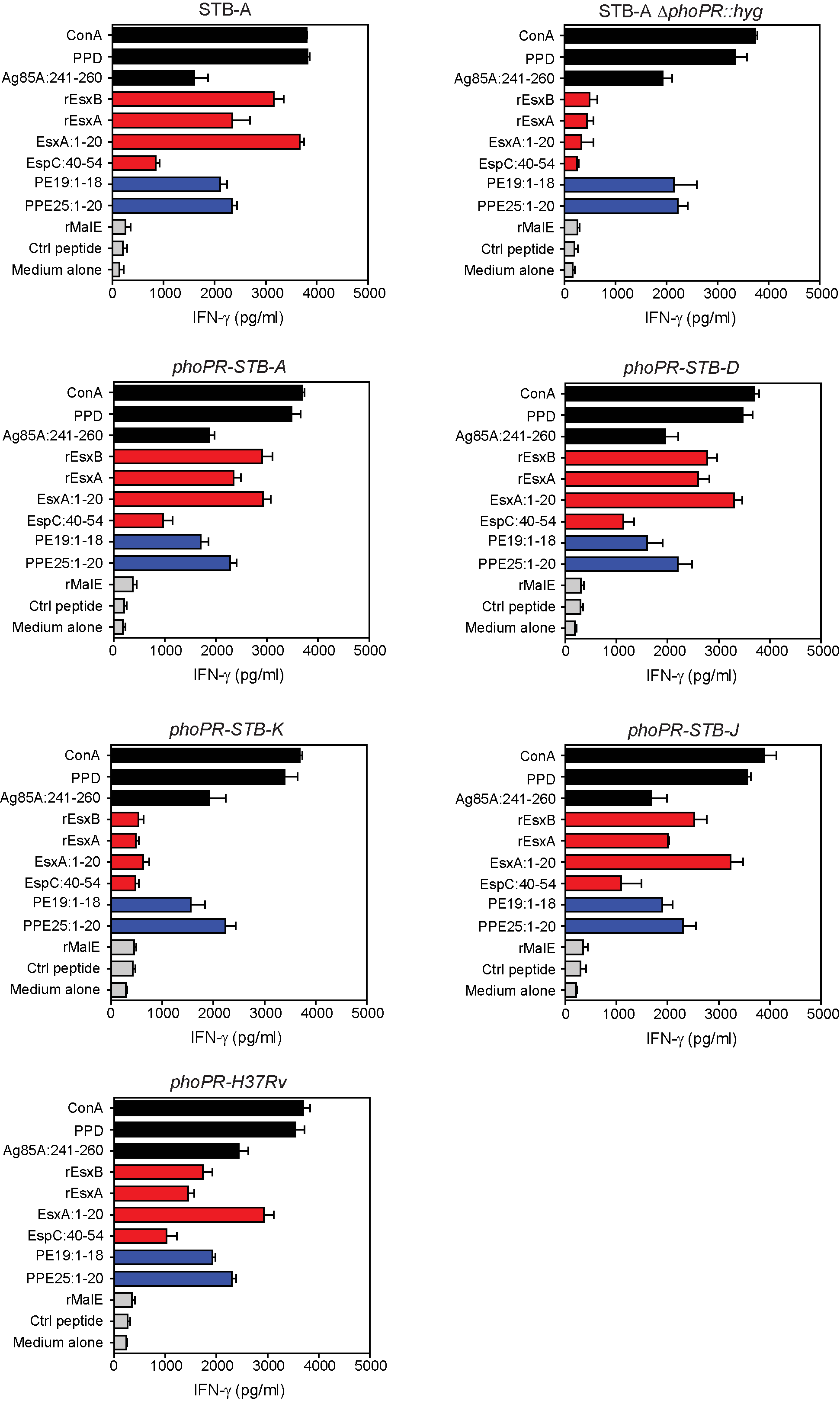

Supplement: S12 Fig — T-cell immune responses of C57BL/6 mice (n = 2 per group) s.c. immunized with 5 x 10^5^ CFU/mouse of different M. canettii mutant or complemented strains. Pool of splenocytes of the immunized mice were cultured ex vivo with various ESX-1 (EsxA, EsxB and EspC) and ESX-5 (PE/PPE) associated antigens during 72 h at 37°C. The IFN-γ was quantified in the culture supernatant by ELISA. Positive controls (black bars) and negative controls (grey bars) were represented. Error bars represent SD. (TIF) [file ppat.1011437.s013.tif]
