# Supplementary material for: Natural mutations in the sensor kinase of the PhoPR two-component regulatory system modulate virulence of ancestor-like tuberculosis bacilli
Source: PLoS Pathog. 2023 Jul 14;19(7):e1011437. doi: 10.1371/journal.ppat.1011437 (PMC10348564; doi:10.1371/journal.ppat.1011437)
Supplement: S2 Table — References are listed in S1 Text. (DOCX) [file ppat.1011437.s015.docx]

**S2 Table:** Name and main features of strains, plasmids, and phages used in this study. References are listed in S1 Text.

| **Name** | **Relevant characteristics** | **Ref./Source** |
| --- | --- | --- |
| **Strains** |  |  |
| H37Rv | *M. tuberculosis* (Lineage 4) | [[4](#_ENREF_4)] |
| HN878 | *M. tuberculosis* (Lineage 2) | [[5](#_ENREF_5)] |
| STB-A | *M. canettii* CIPT140010059 | [[2](#_ENREF_2)] |
| STB-D | *M. canettii* | [[2](#_ENREF_2)] |
| STB-L | *M. canettii* | [[2](#_ENREF_2)] |
| STB-J | *M. canettii* | [[2](#_ENREF_2)] |
| STB-KS | *M. canettii* | [[6](#_ENREF_6)] |
| STB-KR | *M. canettii* | [[6](#_ENREF_6)] |
| H37Rv Δ*phoPR* | H37Rv, Δ*phoPR::hyg* | [[7](#_ENREF_7)] |
| STB-A Δ*phoPR* | STB-A, Δ*phoPR::hyg* | [[7](#_ENREF_7)] |
| STB-KR Δ*phoPR* | STB-KR, Δ*phoPR::hyg* | This study |
|  |  |  |
| **plasmids and phages** |  |  |
| phWM25 | Shuttle phasmid derived from mycobacteriophage TM4 carrying the Δ*phoPR::hyg* substrate for allelic exchange from STB-A | [[7](#_ENREF_7)] |
| pWM222 | Integrative *E. coli*/mycobacteria shuttle vector carrying the *phoPR* genes from *M. tuberculosis* H37Rv (*phoPR-H37Rv*) | [[7](#_ENREF_7)] |
| pWM342 | Integrative *E. coli*/mycobacteria shuttle vector carrying the *phoPR* genes from STB-A (*phoPR-STB-A)* | This study |
| pWM343 | Integrative *E. coli*/mycobacteria shuttle vector carrying the *phoPR* genes from STB-A (*phoPR-STB-D)* | This study |
| pWM344 | Integrative *E. coli*/mycobacteria shuttle vector carrying the *phoPR* genes from STB-A (*phoPR-STB-K)* | This study |
| pWM345 | Integrative *E. coli*/mycobacteria shuttle vector carrying the *phoPR* genes from STB-A (*phoPR-STB-J)* | This study |
| pWM251 | Mycobacterial plasmid derived from pMIP12 containing the *gfp* gene and a streptomycin resistance gene | [[8](#_ENREF_8)] |
