# Supplementary material for: Natural mutations in the sensor kinase of the PhoPR two-component regulatory system modulate virulence of ancestor-like tuberculosis bacilli
Source: PLoS Pathog. 2023 Jul 14;19(7):e1011437. doi: 10.1371/journal.ppat.1011437 (PMC10348564; doi:10.1371/journal.ppat.1011437)
Supplement: S3 Table — (DOCX) [file ppat.1011437.s016.docx]

**S3 Table:** Name and sequences of primers used in this study

| **Name** | **sequences** |  |
| --- | --- | --- |
| ***sigA***  RT-sigA-Fw  RT-sigA-Rv  ***mcr7***  RT-mcr7-canettii-Fw  RT-mcr7-Rv  ***pks2***  RT-pks2-Fw  RT-pks2-Rv  ***lipF***  RT-lipF-Fw  RT-lipF-Rv  ***phoP***  RT-phoP1-KR-Fw  RT-phoP1-KR-Rv  ***phoR***  RT-phoR1-KR-Fw  RT-phoR1-KR-Rv | 5’- CCGATGACGACGAGGAGATC-3’  5’- CGGAGGCCTTGTCCTTTTC-3’  5’- TGACCATGACAGCGAGTGTG-3’  5’- AGGGAGCTGCTTGGACAGAA-3’  5’- GCATCGGTGAAGACCAACTTC-3’  5’- GATTACGTGGAACCACACCATGT-3’  5’- GCAGGCCCGAAGACCTCTAT-3’  5’- GGCGGCAAGCTGGATTC-3’  5’- GCTCGACGAGGAGACCCA-3’  5’- CGATCTTGCGGCGCAGATA-3’  5’- CTGATCCTGGTGGCCACTG-3’  5’- GGATCCGCCGATCGATGG-3’ |  |
|  |  |  |
